# Factors influencing national implementation of innovations within community pharmacy: a systematic review applying the Consolidated Framework for Implementation Research

**DOI:** 10.1186/s13012-019-0867-5

**Published:** 2019-03-04

**Authors:** Natalie M. Weir, Rosemary Newham, Emma Dunlop, Marion Bennie

**Affiliations:** 1 Strathclyde Institute of Pharmacy and Biomedical Sciences, University of Strathclyde, 40 Taylor Street, Robertson Trust Wing, Glasgow, G4 0RE UK; 2 Information Services Division, NHS National Services Scotland, Gyle Square, 1 South Gyle Crescent, Edinburgh, EH12 9EB UK

**Keywords:** Primary care, CFIR, Scale, Roll out, Innovation, Determinant framework, Retail pharmacy

## Abstract

**Background:**

To meet emergent healthcare needs, innovations need to be implemented into routine clinical practice. Community pharmacy is increasingly considered a setting through which innovations can be implemented to achieve positive service and clinical outcomes. Small-scale pilot programmes often need scaled up nation-wide to affect population level change. This systematic review aims to identify facilitators and barriers to the national implementation of community pharmacy innovations.

**Methods:**

A systematic review exploring pharmacy staff perspectives of the barriers and facilitators to implementing innovations at a national level was conducted. The databases Medline, EMBASE, PsycINFO, CINAHL, and Open Grey were searched and supplemented with additional search mechanisms such as Zetoc alerts. Eligible studies underwent quality assessment, and a directed content analysis approach to data extraction was conducted and aligned to the Consolidated Framework for Implementation Research (CFIR) to facilitate narrative synthesis.

**Results:**

Thirty-nine studies were included: 16 were qualitative, 21 applied a questionnaire design, and 2 were mixed methods. Overarching thematic areas spanning across the CFIR domains were pharmacy staff engagement (e.g. their positive and negative perceptions), operationalisation of innovations (e.g. insufficient resources and training), and external engagement (e.g. the perceptions of patients and other healthcare professionals, and their relationship with the community pharmacy). Study participants commonly suggested improvements in the training offered, in the engagement strategies adopted, and in the design and quality of innovations.

**Conclusions:**

This study’s focus on national innovations resulted in high-level recommendations to facilitate the development of successful national implementation strategies. These include (1) more robust piloting of innovations, (2) improved engagement strategies to increase awareness and acceptance of innovations, (3) promoting whole-team involvement within pharmacies to overcome time constraints, and (4) sufficient pre-implementation evaluation to gauge acceptance and appropriateness of innovations within real-world settings. The findings highlight the international challenge of balancing the professional, clinical, and commercial obligations within community pharmacy practice. A preliminary theory of how salient factors influence national implementation in the community pharmacy setting has been developed, with further research necessary to understand how the influence of these factors may differ within varying contexts.

**Trial registration:**

A protocol for this systematic review was developed and uploaded onto the PROSPERO international prospective register of systematic reviews database (Registration number: CRD42016038876).

**Electronic supplementary material:**

The online version of this article (10.1186/s13012-019-0867-5) contains supplementary material, which is available to authorized users.

## Background

A strong primary care system underpins improvements in a nation’s population health [1, 2]; therefore, the primary care sector must continually adapt to meet emergent healthcare needs. Community pharmacies have veered away from traditional dispensing-focused roles as their ability to offer enhanced services within primary care has been recognised [3]. Existing contributions within primary care include the administration of vaccinations [4], smoking cessation support [5], and medication reviews [6, 7]. Additionally, the introduction of pharmacy technicians performing accuracy checks on dispensed medication and the implementation of novel technologies, such as automated dispensing, are considered facilitators to improve efficiency and workflow [8, 9]. This can allow pharmacies more time to offer more patient-focused services.

Access to a healthcare professional without the need for an appointment renders community pharmacies unique to other primary care settings, which enhances the scope of exposure of new services to more patients [10–12]. Successful implementation of innovations within healthcare systems underpins the achievement of intended outcomes—for example, improvements in efficiency, safety, or symptomology [13]. For maximal impact within primary care, and to improve population-level health, innovations need scaled up nation-wide [14].

The complexity of national implementation is well acknowledged [15]. Within community pharmacies, service delivery can be dependent on ownership [16], partly due to the autonomous nature of community pharmacies and their requirement to be profitable. Wide-scale implementation is further complicated as barriers and facilitators identified within small-scale, pilot phases may not be generalisable to the national setting [17]. This may be due to the recruitment of “early adopters” who may be less resistant to change [18, 19].

Two previous reviews have explored implementation within the community pharmacy setting. Organisational and individual facilitators to practice change in relation to cognitive pharmacy services have been identified by Roberts et al. [20]. As few empirical studies at this time explored implementation, the results are mostly centred on hypothetical facilitators. More recently, Shoemaker et al. identified barriers and facilitators to the implementation of three services common in the USA (Medication Therapy Management, immunisations, and rapid HIV testing) [21]. However, methodological approaches adopted by these reviews and associated limitations warrant further exploration within this area. The reviews by Shoemaker et al. and Roberts et al. explored barriers and facilitators only for a subset of innovation types. Neither focused specifically on national innovations and included those limited to pilot stages. Additionally, neither critically appraised the included studies, and the reviews included studies which sought perspectives from individuals with no involvement in implementation, meaning the results may not reflect barriers and facilitators truly experienced in practice.

Considering the evolving role of the community pharmacy setting, and the uniqueness of this context, further exploration is required to understand how innovations in this setting can be scaled up to affect nationwide improvements in health. This systemic review addresses this to identify barriers and facilitators to the national implementation of community pharmacy innovations by building upon the reviews previously conducted and overcoming their associated limitations [20, 21]. The objectives of this systematic review are to:

1. Identify studies exploring the factors influencing the national implementation of community pharmacy innovations from the perspectives of community pharmacy staff
2. Synthesise reported barriers and facilitators
3. Develop recommendations for national implementation strategies

Following completion of the systematic review, the opportunity was recognised to develop a preliminary causal theory of how innovations become successfully implemented within community pharmacies at a national level. Therefore, an additional objective was derived:

4. Develop a causal theory of the factors influencing successful national implementation of community pharmacy innovations

## Methods

This systematic review is presented according to the Preferred Reporting Items for Systematic Reviews and Meta-Analysis (PRISMA) 2009 checklist [22]. A protocol was developed [23, 24] and uploaded onto the PROSPERO register of systematic reviews (Registration number: CRD42016038876) [25].

### Eligibility criteria

Eligible studies sought pharmacy staff’s perspectives on barriers and facilitators to implementing national innovations. An innovation was considered a practice, object, or idea perceived to be new to the setting in which it was implemented [18]. Implementation was considered the process by which an innovation was introduced and applied within the pharmacy setting [26, 27]. Studies solely focusing on views of adoption prior to the implementation of an innovation were outwith the scope of this review as this was considered the preliminary decision-making process of the pharmacy staff to use an innovation [18], which was considered conceptually dissimilar to implementation. Studies involving participants from mixed disciplines (e.g. general practitioners as well as community pharmacy staff) were included if it was possible to extract the data solely pertaining to community pharmacy staff perspectives. Studies from any country were considered eligible for inclusion. Qualitative, quantitative, and mixed method studies were included from peer-reviewed journal articles, conference proceedings, poster presentations, and unpublished literature. The exclusion criteria are presented in Table 1.

**Table 1:** Exclusion criteria

• Studies reporting undefined innovations (e.g. concepts such as “pharmaceutical care”)	
• Studies exploring barriers and facilitators to implementing innovations for specific pharmacy characteristics (e.g. barriers to implementation within independently-owned pharmacies)	
• Studies exploring barriers and facilitators to delivering services to a specific subset of eligible patients (e.g. barriers to delivering medication review services to aboriginal populations specifically)	
• Studies exploring anticipated barriers or facilitators during pre-implementation phases	
• Books, editorials, lecture commentaries, and studies reporting non-original research.	

### Search strategy

The databases Medline, EMBASE, PsycINFO, and the Cumulative Index of Nursing and Allied Health Literature (CINAHL) were searched from their inception on 17 December 2015. Unpublished literature was searched within the Open Grey database [28]. See Additional file 1 for the full Medline search strategy. The search was limited to the English language and covered all studies available up until the search date. Supplementary searches were applied from December 2015 onwards until data analysis concluded in March 2017 (Table 2).

**Table 2:** Supplementary search strategy (December 2015–March 2017)

1. Screening the reference list of included studies	
2. Email alerts from the Zetoc database (a monitoring and search service for global research publications) when new articles were published in the following journals:	
• *Accreditation and Quality Assurance*	
• *BMC Health Services Research*	
• *BMJ Quality and Safety*	
• *Implementation Science*	
• *International Journal for Quality in Health Care*	
• *International Journal of Health Care Quality Assurance*	
• *International Journal of Pharmacy Practice*	
• *International Journal of Quality And Innovation*	
• *Joint Commission Journal on Quality and Patient Safety*	
• *Quality Management in Health Care*	
• *Research in Social and Administrative Pharmacy*	
3. Hand searches of *The Pharmaceutical Journal*	

### Study selection

Titles and abstracts were screened within Covidence [29], with potentially relevant studies progressing onto full-text screening. The primary reviewer (NW) completed the study selection, with a 20% subset of the title/abstracts and full-texts screened independently. A percentage of agreement was calculated and categorised using the following thresholds: < 70%, poor; 70–79%, fair; 80–89%, good; and > 90%, excellent [30]. A percentage of agreement > 80% was considered adequate [31]. Where the data were published in more than one format, the format which underwent the most extensive peer-review process was included (e.g. a journal article would be selected for data extraction over a conference proceeding).

### Data extraction

A data extraction table was devised [24] and piloted in approximately 10% of studies. Piloting identified that delineating the data to barriers and facilitators was oversimplistic as the studies also reported on suggestions of what would have facilitated implementation. These were termed “hypothetical facilitators” and were extracted separately.

### Quality assessment

Quality assessment tools were used specific to the method(s) employed. This was to ensure that existing purposefully designed tools were used to assess the quality of either qualitative, quantitative, or mixed method studies to comparable depth. They consisted of a series of questions exploring aspects such as the clarity of the aim, appropriateness of the methodology, recruitment of participants, and data analysis. The 34-item Critical Appraisal Skills Programme (CASP) tool was used to appraise qualitative studies [32]. For questionnaire design studies, the Boynton and Greenhalgh Quality Checklist (BGQC) tool was used [26]. The mixed method studies all applied interviews alongside a questionnaire. Existing mixed method quality assessment tools did not assess questionnaires to the same depth as the BGQC tool [33] and would not offer comparable quality assessment. Therefore, the mixed method studies were assessed using the initial screening questions within the Mixed Methods Appraisal Tool (MMAT) [34], which explores the appropriateness of the mixed method approach, with each method then assessed by the CASP or BGQC tool [26, 32].

The quality assessment tools each have screening questions on the clarity of the aim and appropriateness of the research design. Studies were excluded if these initial criteria were not met. Questionnaire studies which used only closed-ended questions were excluded unless based on previous qualitative work or wider literature as the researchers would have introduced bias based on their a priori assumptions of influential factors [35]. To generate the quality assessment result for each study, each question within the quality assessment tool was attributed a score of 2 if the study fully met the criteria, 1 if partially met, and 0 if not met or unclear. The quality assessment results are presented as percentages as not all questions were applicable to every study [32, 36]. The quality assessment results for the mixed method studies were calculated from the lowest scoring method to ensure the final result did not exceed the quality of the studies weakest component [34]. The quality assessment was conducted by the primary reviewer (NW), with clarification from a mediator when required (ED).

### Synthesis of results

The Consolidated Framework for Implementation Research (CFIR) was selected to synthesise the data [37]. The CFIR is a determinant implementation framework of factors influencing implementation amongst five domains: intervention characteristics, the inner setting, the outer setting, characteristics of the individual, and the implementation process [38]. It is widely applied [39], commonly to explore healthcare practitioners’ experiences of implementing an innovation [40], which facilitates cross comparison of results [41]. Each CFIR domain has a number of constructs which are defined in Additional file 2.

A directed content analysis approach [42] was applied where data extraction was conducted inductively, with the synthesis afterwards deductively aligned to the CFIR [43, 44]. This allowed data capture of barriers and facilitators not within the CFIR to test its applicability within the community pharmacy context. As the CFIR constructs are conceptually broad (e.g. one construct is “Knowledge and Beliefs”), data within each CFIR construct was explored for emergent subconstructs [45]. A table quantifying the emergence of barriers, facilitators, and hypothetical facilitators within each CFIR construct was developed, with overarching thematic areas identified from visual analysis [45]. A descriptive narrative synthesis method was chosen to present commonly reported CFIR constructs to facilitate integration of qualitative and quantitative results [46–48].

To examine the robustness of the results, a sensitivity analysis involved removal of studies with a quality assessment result of < 50% to observe what effect this had on the reporting frequency of the barriers, facilitators, and hypothetical facilitators [49]. As different studies evaluated the same innovation, the results were categorised both by study and by innovation to see how this affected reporting frequency.

## Results

### Study selection

**Fig. 1:**
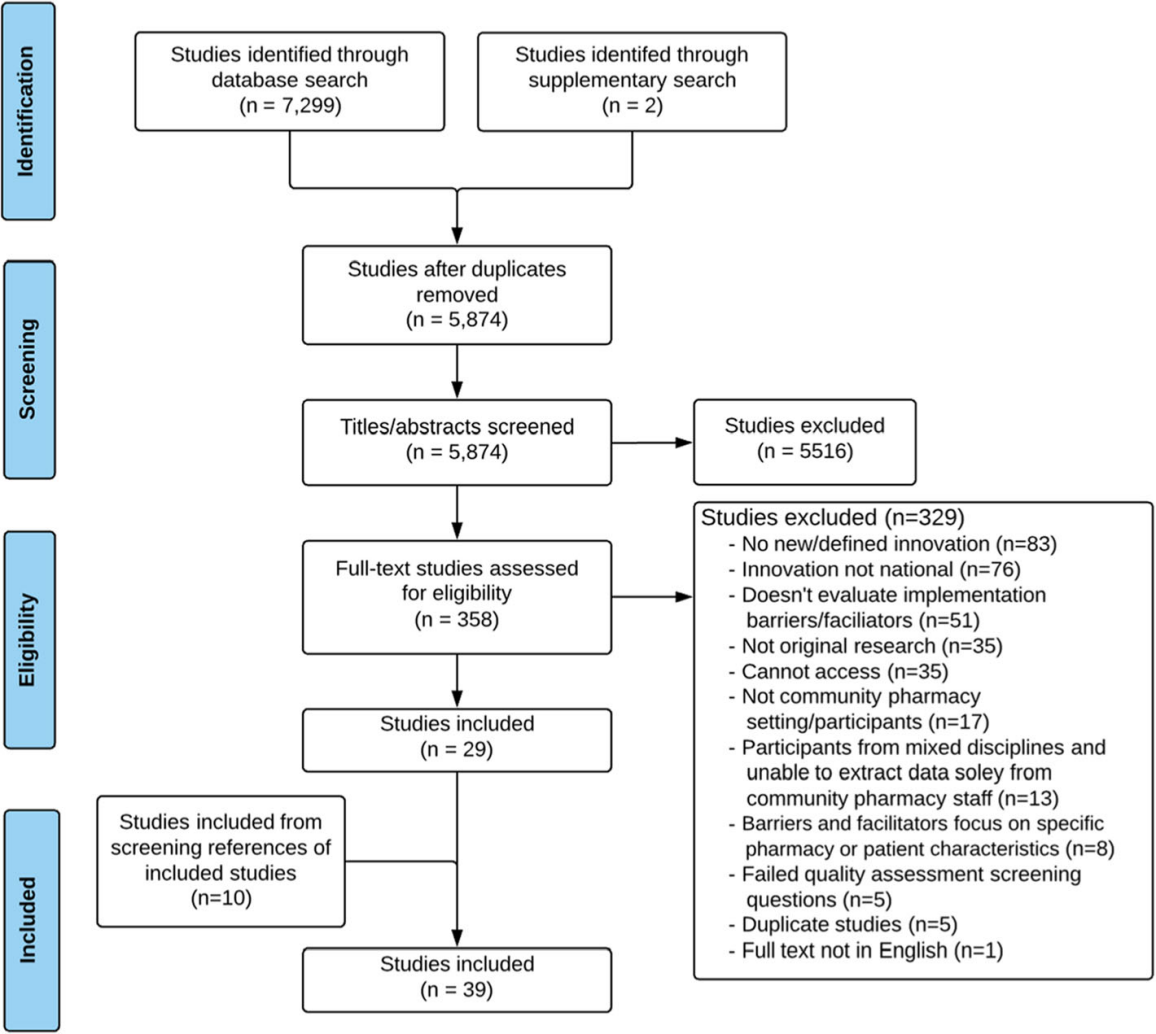
Flow chart of screening process to identify relevant studies (December 2015–March 2017)

Thirty-nine studies were included from the 5874 studies which had titles and abstracts screened (Fig. 1) [6, 50–87]. The percentage of agreement of the titles and abstracts independently screened was 94% (excellent), and for full-texts was 88% (good).

### Study characteristics

All studies were published from 2002 onwards, and most since 2010 (*n* = 28, 72%). Approximately half (*n* = 20, 51%) originated from the UK. Ten studies originated from other European countries, and the other nine were from Australia (*n* = 3), Malaysia (*n* = 2), New Zealand (*n* = 2), Saudi Arabia (*n* = 1), and Cambodia (*n* = 1). The innovation types can be categorised into four subtypes: clinical service (*n* = 21); pharmacovigilance (*n* = 6); e-technology (*n* = 2); and legislative change (*n* = 10) such as policy changes and reclassification of medicines. Some studies evaluated the same innovation: the UK “Healthy Living Pharmacy” framework (*n* = 5), the UK “New Medicines Service” (n = 3), the “Danish’ Inhaler Technique Assessment Service” (*n* = 2), the UK “Medicines Use Review” (*n* = 3), the Malaysian spontaneous adverse drug reporting system “MADRAC” (*n* = 2), and the Swedish implementation of ePrescribing (*n* = 2). Resultantly, the 39 studies report on 28 innovations. Excluding one study which did not provide participant numbers [69], the total number of participants included from the studies is 12,172. Only ten studies (26%) explored perspectives of community pharmacy support staff [52, 54, 57, 61, 63, 69, 70, 75, 82, 83], and two did so exclusively [57, 82]. The full characteristics of included studies are within Additional file 3.

### Quality assessment results

An overview of the quality assessment results is within Table 3, with the full quality assessment in Additional file 4. Five studies were excluded as they applied questionnaires with only closed-ended questions which were not reported to be developed from reference to literature or previous qualitative findings [88–92].

**Table 3:** Summary of quality assessment results of included studies (*n* = 39)

Qualitative studies (*n* = 16)	Result (%)	Questionnaire design studies (*n* = 21)	Result (%)	Mixed method studies (*n* = 2)	Result (%)
Chaar 2013^*^ [55]	74	Chee Ping 2010^*^ [80]	79	Thomas 2009^^^[84]	25
Lucas 2015^*^ [77]	71	Duarte 2015^*^ [58]	76	Blenkinsopp 2007^^^[53]	19
Donovan 2016^*^ [57]	70	Bawazir 2006^*^ [51]	74		
Rutter 2015^*^ [82]	68	Hamrosi 2014^*^ [65]	70		
Kaae 2010^*^ [69]	66	Elkalmi 2014^*^ [59]	68		
Bell 2012^*^ [52]	65	Paudyal 2010^*^ [79]	68		
Kaae 2011^*^ [69]	65	Kansanahoa 2005^*^ [71]	65		
Firth 2015^*^ [61]	63	Paudyal 2012^*^ [78]	64		
Gauld 2011^*^ [62]	63	Weidmann 2011^*^ [86]	64		
Elkalmi 2011^*^ [60]	62	Hansford 2007^*^ [66]	64		
Latif 2016^*^ [74]	62	Hammar 2010^*^ [64]	61		
Brooks 2013^*^ [54]	47	Van Grootheest 2002^*^ [85]	62		
Wilcock 2008^*^ [87]	42	Irujo 2007^*^ [68]	57		
Longergan 2012^^^ [75]	41	Latif 2008^*^ [72]	55		
Shevket 2015^^^ [83]	35	Rahimi 2011^*^ [81]	50		
Corlett 2013^^^ [56]	33	Gröber-grätz 2010^*^ [63]	50		
		Lee 2008^*^ [6]	48		
		Allenet 2003^*^[50]	38		
		Latif 2010^^^ [73]	37		
		Loo 2011^^^ [76]	31		
		Hodson 2014^^^ [67]	29		

^*^Peer-reviewed journal paper

^^^Conference abstract

#### Qualitative studies

All qualitative studies (*n* = 16) detailed the research aims and rationalised the study’s importance and relevance. Six justified why a qualitative design was chosen [54, 55, 60, 70, 77, 83], and three justified the specific method adopted [61, 69, 77]. For one study which employed two qualitative methods, only one method was explicitly justified [74]. Six studies made the methods fully explicit by including the interview or focus group guide [52, 55, 57, 61, 74, 77]. No studies offered a full account of reflexivity as none adequately considered the researcher-participant relationship, although some reflected on potential bias during data collection and sampling [57, 62, 77, 82]. No study sufficiently discussed the credibility of their findings as per the quality assessment criteria.

#### Questionnaire design studies

All questionnaire design studies (*n* = 21) had a clear research question. Six studies did not attain a response rate of > 50% [59, 65, 76, 78, 79, 86], and three employed sampling methods which made it not possible to determine response rates [50, 63, 80]. Two studies sampled a single pharmacy chain [72, 73], and six sampled participants within specific geographical locations [51, 58, 59, 63, 68, 81]. Twelve studies piloted the questionnaire in a representative cohort [6, 51, 58, 59, 64–67, 78–80, 86]. Four studies did not offer sufficient detail to determine if the pilot sample was representative of study participants [50, 63, 68, 85], and for one study, the pilot sample was not representative [72]. One study modified an existing questionnaire without re-piloting it [73], and three studies did not state if the questionnaire was piloted [71, 76, 81]. Three studies had claims for both validity and reliability [51, 59, 80], ten had claims for neither [6, 50, 58, 63, 65, 67, 68, 71, 73, 85], and the remaining eight conducted face and/or content validity testing [64, 66, 72, 76, 78, 79, 81, 86].

#### Mixed method studies

The mixed method studies were low in quality due to insufficient details as both were conference proceedings [53, 84]. One study did not explain the rationale for integrating qualitative and quantitative methods [53], and neither described how the data was integrated [53, 84].

### Barriers, facilitators, and hypothetical facilitators

The reporting frequency of the barriers, facilitators, and hypothetical facilitators aligned to the CFIR constructs is shown in Table 4. A full presentation of constructs and subconstructs is in Additional file 5. When removing studies with a quality assessment score of < 50% (*n* = 12, 41%), no changes were identified to the most commonly reported CFIR constructs amongst the barriers, facilitators, and hypothetical facilitators. Likewise, when categorising results by innovation and not by study, the most commonly reported CFIR constructs remained the same. Therefore, for completeness, all studies were retained within the analysis.

**Table 4:** Frequency table of cited Consolidated Framework for Implementation Research (CFIR) constructs (*n* = 39 studies)

CFIR domains (*n* = 5) and constructs (*n* = 39)	Barrier *n* (%) of studies	Facilitator *n* (%) of studies	Hypothetical facilitator *n* (%) of studies
Intervention Characteristics			
Intervention source	0 (0)	0 (0)	0 (0)
Evidence strength and quality	1 (3)	0 (0)	0 (0)
Relative advantage^*^	7 (18)	12 (31)	0 (0)
Adaptability	7 (18)	1 (3)	2 (5)
Trialability	0 (0)	0 (0)	0 (0)
Complexity^*^	12 (31)	2 (5)	3 (8)
Design quality and packaging^*^	10 (26)	2 (5)	11 (28)
Cost	6 (15)	0 (0)	0 (0)
Outer setting			
Patient needs and resources^*^	21 (54)	9 (23)	0 (0)
Cosmopolitanism^*^	15 (38)	4 (10)	5 (13)
Peer pressure	0 (0)	0 (0)	1 (3)
External policy and incentives^*^	6 (15)	2 (5)	11 (28)
Inner setting			
Structural characteristics	0 (0)	3 (8)	0 (0)
Networks and communications	2 (5)	2 (5)	0 (0)
Culture	0 (0)	0 (0)	0 (0)
Implementation climate			
Tension for change	1 (3)	0 (0)	0 (0)
Compatibility^*^	9 (23)	12 (31)	1 (3)
Relative priority	3 (8)	0 (0)	0 (0)
Organisational incentives and rewards^*^	1 (3)	15 (38)	0 (0)
Goals and feedback	2 (5)	2 (5)	6 (15)
Learning climate	0 (0)	0 (0)	0 (0)
Readiness for implementation			
Leadership engagement	3 (8)	2 (5)	0 (0)
Available resources^*^	28 (72)	7 (18)	10 (26)
Access to knowledge and information^*^	8 (21)	5 (13)	17 (44)
Characteristics of individuals			
Knowledge and beliefs about the intervention^*^	22 (56)	21 (54)	0 (0)
Self-efficacy	4 (10)	6 (15)	0 (0)
Individual stage of change	6 (15)	9 (23)	0 (0)
Individual identification with organisation	0 (0)	0 (0)	0 (0)
Other personal attributes	4 (10)	5 (13)	0 (0)
Process			
Planning	1 (3)	0 (0)	3 (8)
Engaging			
Engaging stakeholders^*^^	1 (3)	0 (0)	12 (31)
Engaging innovation participants^*^^	3 (8)	1 (3)	12 (31)
Opinion leaders	0 (0)	0 (0)	0 (0)
Formally appointed internal opinion leaders	0 (0)	0 (0)	0 (0)
Champions	0 (0)	0 (0)	0 (0)
External change agents	0 (0)	0 (0)	0 (0)
Executing	0 (0)	0 (0)	1 (3)
Reflecting and evaluating	0 (0)	0 (0)	1 (3)

^*^Represents CFIR constructs cited by at least ten studies (25%) as a barrier, facilitator, or hypothetical facilitator

^^^The CFIR construct “Engaging” has been subdivided into “Engaging Stakeholders” and “Engaging Innovation Participants” as per the CFIR qualitative codebook guidelines (https://cfirguide.org/constructs/engaging/)

Thirteen (33%) CFIR constructs were cited by at least ten studies (25%) as a barrier, facilitator, or hypothetical facilitator. Overarching thematic areas spanning across the CFIR domains were identified: pharmacy staff engagement, operationalisation of the innovation, and external engagement (Table 5).

**Table 5:** Overarching thematic areas identified from included studies (*n* = 39) across commonly reported Consolidated Framework for Implementation Research (CFIR) constructs

Thematic areas	Description	CFIR construct (CFIR domain)
Pharmacy staff engagement	Pharmacy staff’s knowledge and beliefs relating to an innovation, its compatibility with their roles and values, whether it poses advantages or not, and the incentives and strategies which engage community pharmacy staff.	• Knowledge and beliefs about the intervention (characteristics of individuals) • Compatibility—with roles or values (inner setting)^*^ • Relative advantage (innovation characteristics) • External policy and incentives (outer setting) • Organisational incentives and rewards (inner setting) • Engaging innovation participants (process)
Operationalisation of the innovation	Innovation attributes (such as design and complexity) and surrounding factors including resources, compatibility with pharmacy systems, and pharmacy staff access to knowledge and information about the innovation.	• Available resources (inner setting) • Design quality and packaging (innovation characteristics) • Complexity (innovation characteristics) • Compatibility—with systems (inner setting)^*^ • Access to knowledge and information (inner setting)
External engagement	The relationship with patients and other healthcare professionals, their perceptions, and strategies to engage these stakeholders.	• Cosmopolitanism (outer setting) • Patient needs and resources (outer setting) • Engaging stakeholders (process)

^*^The compatibility construct of the CFIR was delineated into “Compatibility—with roles and values” and “Compatibly—with systems”

#### Pharmacy staff engagement

##### Knowledge and beliefs about the intervention

Positive and negative views of the innovation by the pharmacy staff were commonly cited factors influencing implementation. Nine studies had mixed views from participants [52, 55, 57, 62, 72, 73, 78, 85, 86]. The negative perceptions were varied and in many instances context specific, but included concerns and a lack of belief in/support for the innovation [51, 52, 55, 57–60, 62, 66, 69, 72–74, 78, 80, 85–87]. Positive perceptions included a belief in/support for the innovation [6, 50–52, 55–62, 67, 68, 72, 73, 75, 78, 85, 86] and positivity about the way the innovation was implemented [84].

In four studies, good awareness and understanding surrounding the innovation were cited [52, 62, 68, 85]. Notably for two of these, lack of knowledge was also cited by some participants [62, 85]. Lack of awareness [51, 59, 60, 68, 84] and operational knowledge [51, 57–60, 62, 68, 76, 84, 85] was common, and lack of appropriate clinical knowledge was cited by five studies [51, 58, 59, 70, 85]. All pharmacovigilance studies cited a lack of awareness and knowledge [51, 58–60, 68, 85].

##### Compatibility—with roles/values

An innovation’s compatibility with the roles and values of a pharmacist was reported. This included alignment with ambitions [78], the innovation recognising the potential of the pharmacy profession to adopt enhanced or professional roles [61, 74], or being considered integral to a pharmacist’s role [51, 56, 59, 60, 68, 85, 87].

##### Relative advantage

An innovation offering an advantage was evident within 12 studies [50, 54, 61, 64, 75, 77, 79–81, 83, 85, 87]. General advantages included enhanced engagement or relationship with patients [50, 61, 79, 87]; improvement in workforce capability, such as improved education, awareness, or confidence [54, 61, 75, 85]; better relationship with surrounding healthcare professionals [79, 83]; and the innovation benefitting patients [64, 75, 77, 79, 80, 83]. Some were context specific, for example, the time-saving aspect of the Swedish ePrescribing system [64, 81], and the Scottish Minor Ailments Service [79] meaning product cost is no longer considered during consultations [79]. Six studies reported that the innovation presented disadvantages [55, 62–64, 79, 81], and three cited both advantages and disadvantages of an innovation [64, 79, 81].

##### External policy and incentives

This construct relates to policy and incentives originating from government or other central entities [93]. In relation to external policy, reported barriers included the innovation not being aligned with policy [65]. Studies suggested hypothetical facilitators including extending the scope of innovations [6, 52, 65] and making participation in pharmacovigilance innovations compulsory [51, 58, 60, 85]. In relation to external incentives, lack of/insufficient funding [6, 83] or remunerations [50, 76, 79] were reported barriers. Suggestions of incentives which could facilitate implementation were primarily financial [50–52, 60, 65, 72, 76, 85], but also included the provision of awards, certifications, journal subscription, and conference attendance [60] or penalising other healthcare professionals for lack of co-operation [50].

##### Organisational incentives and rewards

This CFIR construct relates to incentives and rewards originating from specific pharmacy organisations, as well personal incentives of the community pharmacy staff [93]. One study cited negative perceptions of target setting within the pharmacy, which were perceived as income-focused rather than patient-focused [87]. For clinical services and legislative changes, personal rewards included improved professional recognition [50, 56, 61, 79], enhanced influence or role [50, 72, 77–79, 86], and personal or professional satisfaction [52, 56, 74, 76, 82]. Commercial benefits spanned across all innovations, including financial betterment for the pharmacy [57, 61, 64, 78, 79] and increased customer footfall [52, 83].

##### Engaging innovation participants

There was little data pertaining to how the implementation strategy influenced implementation. Better informing and engaging the pharmacy workforce was a suggested hypothetical facilitator [51, 54, 55, 59, 60, 68, 71, 74, 85], including better collaboration between pharmacies [54], educational campaigns [71], mentoring and networking opportunities [55, 74], culture change [68], better advertising [51, 85], and promotion [59, 60, 85].

#### Operationalisation of the innovation

##### Available resources

Lack of time [6, 50–54, 57–61, 65, 68, 70–73, 75, 76, 79, 81, 83, 85, 87] and increased workload [50, 54, 61, 83, 84] associated with an innovation were common. Issues included insufficient staffing [72, 73, 76, 78] and losing staff to training events [61], and one study reported the benefits of having two pharmacists on duty [74]. Two studies reported a general lack of operational resources [70, 79]. Studies mostly reported innovation-specific barriers such as a lack of printers [65] and reporting forms for adverse drug reporting [51, 58–60, 85]. Lack of access to clinical information was cited for both the legislative and clinical services [66, 67, 72, 78, 79] as was a lack of suitable space [57, 72, 76, 78]. Two studies cited adequate pharmacy facilities, such as a consultation room [71, 73]. Six studies reported valued resources associated with an innovation [57, 61, 62, 64, 71, 74], and nine suggested resources which would facilitate implementation [6, 51, 55, 65, 72, 79, 81, 84, 87], including access to clinical information about patients [79, 87], reporting forms [50], improved IT systems [6, 55, 65, 79, 81], a consultation area [71], and a “fact sheet” to facilitate implementation [84].

##### Design quality and packaging

Poor design was most common amongst clinical services. This included the requirement of patient consent devaluing the innovation and patient’s perception of the pharmacist as a professional [56], being unaware of a patient’s discharge from hospital [67], lapsing of customer registration [79], and inappropriate patient referrals [55]. For a service where medication information was legislated to be supplied to all patients, information was not available in other languages and considered too long to print [65]. For a pharmacovigilance program, centralisation of the reporting system was a barrier [59]. Poor quality of the innovation mainly pertained to IT or system issues [58, 61, 74, 78, 81] or the nature of the paperwork involved [58, 67]. Eleven studies suggested improvements to the design and quality of the innovations [51, 55, 58–60, 64, 65, 67, 72, 85, 86].

##### Complexity

Difficultly of an innovation [50, 56, 57, 63, 74, 78] and the complexity of its operationalisation were reported [60, 78], with the latter most commonly relating to the complexity of the remuneration or reporting processes [6, 51, 56, 59, 74, 85]. One study reported barriers relating to the complexity of the implementation process itself [84]. Three studies suggested simplifying reporting procedures [51, 59, 85].

##### Compatibility—with systems

Incompatibility with work systems [65] or applicability in certain settings [57] was reported. Three studies cited compatibility with working systems as a facilitator [57, 71, 74], and work process change was suggested in one study to facilitate [65].

##### Access to knowledge and information

Two studies cited training received to be useful [57, 86]; however, a lack of appropriate training was cited by other participants for one of these studies [57]. Inadequacy of the training was cited by five studies, including the training not meeting the needs of staff [86], the lack of appropriate training [55, 57, 71], or the training focusing on filling out forms rather than skills-based [87]. Whilst three studies cited a lack of information available on the innovation [57, 74, 79], four had participants comment positively on information received [57, 62, 71, 78]. Better access to information and training was a suggested hypothetical facilitator reported by 17 studies [50, 51, 55, 57–60, 62, 65, 68, 71, 74, 76, 77, 80, 84, 85], including suggestions of continuous training [50, 51, 59, 60, 62], mentoring and peer review [74], and incorporating training within undergraduate pharmacy degrees [58, 60, 85].

#### External engagement

##### Cosmopolitanism

Pharmacy staff perceived that other healthcare professionals held negative views for the legislative and clinical services. Seven studies cited negative response [6, 50, 52, 72, 74, 77, 79], which included reluctance [50], lack of support [78], or general negative views [6, 52, 72, 74, 77]. Lack of referral was a cited barrier for clinical services [54, 55, 67, 77], and lack of collaboration and communication with healthcare professionals was also apparent [60, 71, 74, 77–79, 87]. Facilitators included doctor referrals [61], establishment of new contacts [54], and having pre-existing relationships with other healthcare professionals [77].

##### Patient needs and resources

Although there were no reports from studies evaluating pharmacovigilance innovations within this construct, other innovation types had numerous citations. Patient’s support and acceptance of the innovation [75, 77, 80, 83] and positive feedback [54, 57, 82, 83] were contrasted by negative perceptions [50, 52, 63, 75, 77, 78]. These included resistance to change or advice [50, 63, 75, 78], perceiving the innovation as lacking in value [77], and perceiving “pharmacists as drug suppliers only” [52]. Two studies reported patient demand [55, 57]; however, pharmacy staff generally reported low public demand [57, 62, 74, 77–79, 86] or that patients were uninterested or reluctant [6, 65, 72, 77]. For the clinical services, there was difficulty recruiting patients [56], reaching targets [61, 77], and retaining patients [56], or patients could not attend appointments [53, 74, 77]. One study reported public awareness [57], yet lack of public knowledge or awareness was more commonly reported [54, 57, 61, 63, 64, 74, 77, 82].

##### Engaging stakeholders

Although three studies cited lack of advertising or promotion of the innovation as a barrier to patient engagement [59, 74, 79], 12 studies across all innovation types suggested better informing and engaging patients [6, 50, 52, 54, 57, 61, 64, 65, 68, 76, 80, 85]. One study reported that banners and displays increased customer awareness [83]. Suggested facilitators included patient education programmes [64, 65, 79, 80] and local publicity and media campaigns [52, 54, 57, 61]. Five studies suggested better engagement with other healthcare professionals [50, 64, 68, 74, 77].

## Discussion

This systematic review evaluated heterogeneous innovations to identify the factors influencing national implementation within the community pharmacy setting. Three key thematic areas were identified: (1) pharmacy staff engagement with implemented innovations, including staff perceptions and beliefs regarding the innovation; (2) operationalisation of the innovation, such as lack of resources; and (3) external engagement with implemented innovations, including perceived negative views of patients and other healthcare professionals. We discuss each in turn with cross comparison to previous reviews conducted by Roberts et al. [20] and Shoemaker et al. [21]. A comparison of the emergent CFIR constructs with these reviews is available in Additional file 6.

In relation to pharmacy staff engagement, mixed positive and negative perceptions of innovations by pharmacy staff were apparent. This is in contrast to previous reviews which found that pharmacists are generally favourable towards new services [94] and that pharmacists’ positive beliefs and attitudes about a service facilitate successful implementation [20, 21]. This review’s findings may reflect the inclusion of national innovations only, as barriers emerging in small-scale pilot phases may not reflect the barriers experienced following scale up [17].

Innovations were considered advantageous by pharmacy staff if they enhanced the relationship with patients, improved relationships with surrounding healthcare professionals, benefitted patients, or improved workforce capability. Personal incentives included professional satisfaction, recognition, and influence. Shoemaker et al.’s review also identified that improving patients’ health and relationship with the pharmacy was advantageous [21] and that demonstration of skillset and perceived value to the public was an incentive [21]. Shoemaker at al additionally found the acquisition of new patients attending the pharmacy to be a facilitator of implementation [21], and the positive influence of monetary incentives and financial betterment mirrors previous findings [20, 21]. These findings highlight the challenge of balancing the professional, clinical, and commercial obligations within the community pharmacy setting [95]. Although exploring pre-implementation phases was outwith the scope of this review, exploring the cognitive processes underpinning decisions to implement innovations in light of financial and personal incentives, and how these weigh against patient-related benefits, would be an interesting area for future research [96].

The most commonly reported barrier in relation to the operationalisation of an innovation was the lack of available resources (e.g. time and workload constraints) which echoed Shoemaker et al.’s findings [21]. Beyond staff recruitment, the promotion of whole-team engagement and delegation of tasks to pharmacy support staff may facilitate more efficient workflow [97–101] and practice change [20]. Barriers relating to insufficient resources and training were common, as was poor design, complexity, and incompatibly of the innovation, with the latter two also identified by Shoemaker et al. [21]. “Bottom-up” implementation strategies with front-line staff involved in the design and testing of innovations may overcome resource insufficiencies and design flaws [102, 103].

External engagement centred on the perceptions of other healthcare professionals and patients. Negative perceptions of other healthcare professionals, and lack of communication and collaboration with pharmacy staff, was a barrier. Roberts et al. also identified that communication with doctors and their attitudes influenced successful implementation of cognitive pharmacy services [20], whilst Shoemaker et al. identified cosmopolitanism and engagement of the wider healthcare setting to be a facilitator [21]. General practitioners have previously reported lack of collaboration with community pharmacies [104], with evidence that they are cautious about their adoption of new services [105] and clinical roles [106–109].

The influence of perceived patient acceptance was also prominent—community pharmacy staff cited lack of patient demand and their resistance towards innovations. Conversely, a review of patient-reported satisfaction with community pharmacy services found high satisfaction [110], and a disparity between how pharmacists perceive patient satisfaction and how patients report satisfaction has been previously identified [111]. Shoemaker et al. identified patient demand for vaccination services and acceptance of Medication Therapy Management, with no barriers identified relating to patient engagement [21]. Additionally, low public awareness of innovations was commonly cited, and there have been mixed findings in relation to patient awareness of community pharmacies’ roles [111–115]. Informing the public was a commonly reported hypothetical facilitator, suggesting that poor public engagement is perceived as a limitation of the implementation strategies adopted.

### Development of a preliminary theory and recommendations for future research

**Fig. 2:**
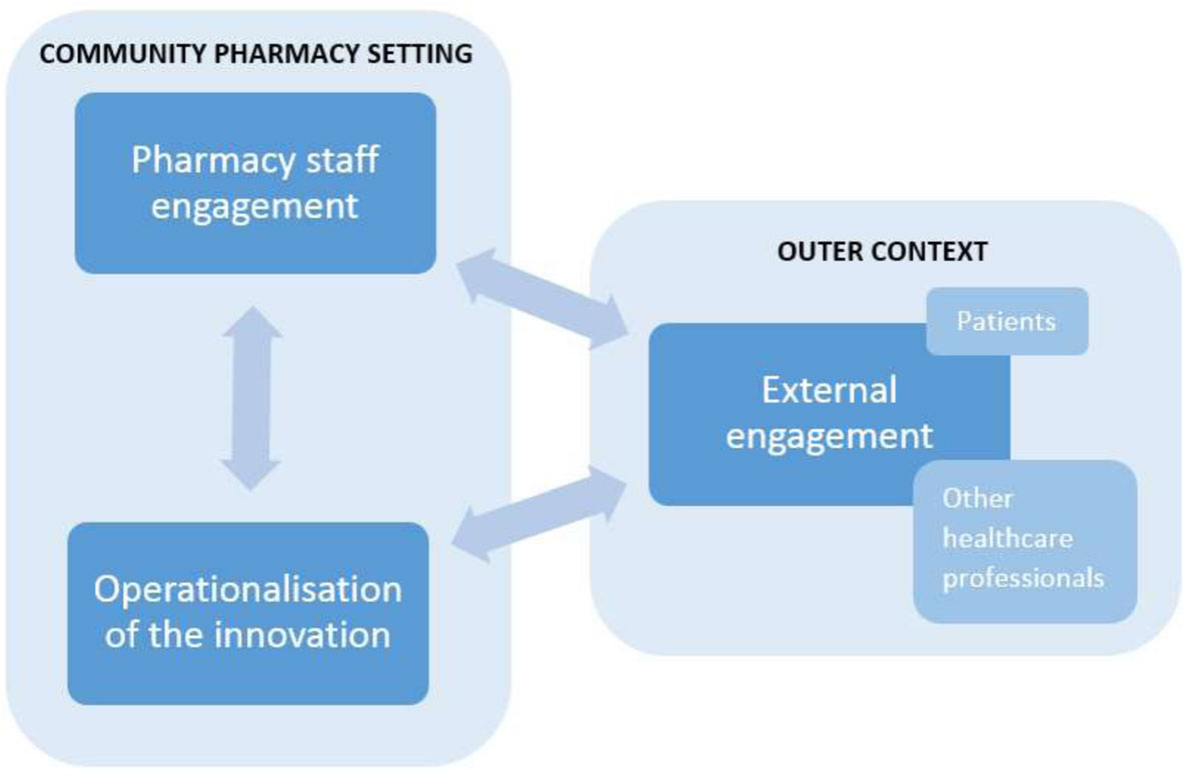
Preliminary theory of the influences affecting the national implementation of community pharmacy innovations

A critical output of this systematic review was the identification of the three overarching thematic areas (Table 5). The identification of these overarching thematic areas allowed for a preliminary causal theory to be developed (Fig. 2). This was not an initial objective of this review as per the initial PROSPERO record (Registration number: CRD42016038876). This preliminary theory proposes that successful national implementation of an innovation requires (1) the pharmacy staff to positively engage with an innovation, (2) the innovation to be easily operational within community pharmacies, and (3) the positive external engagement of patients and other healthcare professionals. The authors view a community pharmacy-specific theory to be necessary due to its distinctiveness when compared to other primary care settings—for example, patients are able to consult with a healthcare professional within community pharmacies without the need for an appointment. Furthermore, the theory presents the high-level influencers of successful implementation within this setting, which may facilitate evaluations if exploration of all 39 CFIR constructs is not feasible.

Further work is needed to understand the interaction between the three overarching thematic areas and to consider how the influence of each may differ within varying contexts and settings. Once tested further, applying this theory at an early stage of an innovation’s implementation process could inform potential refinements to an innovation. As “External Engagement” was an emergent theme, this exemplifies the impact of the surrounding outer context and indicates that the community pharmacy setting is not detached from the wider primary care setting. Wider exploration of the perspectives of other healthcare professionals and the public may strengthen this understanding.

In line with previous work, this review identified that adopted implementation strategies are poorly reported in the literature [14, 21], and the development of innovations was also poorly described. Future studies should explicitly report the implementation strategies being adopted and greater details of the innovation being implemented, including its development, to allow for consideration of how these aspects may influence successful implementation [116, 117].

The emergent CFIR constructs identified in this review complemented those from related reviews [20, 21], which suggests our findings are valid. However, given the methodological differences in the review approaches, this should be interpreted with a degree of caution. Two CFIR constructs cited by Shoemaker et al. and Roberts et al. were not emergent in this review: “Culture” [20, 21] and “Trialability” [21]. As Roberts et al. only reported “culture in pharmacy” and did not elaborate, it was not possible to consider why culture was reported in this previous review and not in the current review. Shoemaker et al. coded “alignment with missions/priorities” and “commitment to providing preventive services” within “Culture”, which the current authors would have coded within “Compatibility” (i.e. how the innovation “aligns with individuals’ own norms and values”) [37]. With regards to “Trialability”, Shoemaker et al. identified that slow expansion of immunisation services facilitated implementation, which would unlikely be salient within scaled-up national services [21]. It was more common for differences to emerge in the valence of the reported CFIR constructs. For example, the current review identified negative patient perceptions (patient needs and resources) and lack of engagement of external healthcare professional (cosmopolitanism), whereas Shoemaker et al. only reported facilitators within these CFIR constructs [21]. This may suggest that emergence of barriers and facilitators at national level is comparable to those in small-scale pilot stages, yet the strength of influence they have on implementation may differ, which would be an interesting area for further research.

### Recommendations for future implementation strategies

From these findings, future implementation strategies (Table 6) for national community pharmacy innovations should involve the use of piloting strategies, promotion of whole-team involvement, pre-implementation exploration, and stakeholder engagement strategies.

**Table 6:** Key recommendations for future national implementation strategies within the community pharmacy setting

1. Conduct more robust piloting of innovations to overcome operational issues, for example, using “bottom-up” techniques. Phased implementation strategies may facilitate scale-up, whereby innovations are tested and iteratively re-designed in gradually larger settings. This could ensure innovations are ready for mass application by testing their feasibility and appropriateness in different contexts [17, 116, 117].	
2. Promote whole-team involvement with innovations to overcome resource barriers such as time and workload constraints.	
3. Conduct pre-implementation exploration to identify training needs, and to predict pharmacy staff acceptance of innovations by considering if the innovation poses any of the advantages and incentives identified within this review.	
4. Develop more thorough stakeholder engagement strategies to overcome barriers relating to acceptance of external healthcare professionals and raise general public awareness of innovations and acceptance through emphasis of intended benefits.	

### Strengths and limitations

Alignment of the results to the CFIR positions the research within the wider implementation science literature. However, the inclusion of qualitative and quantitative studies and the varying level of reporting detail meant it was not possible to weight identified barriers and facilitators and deduce relative influences. The results instead reflected the number of reporting studies, which is not uncommon for reviews of this nature [20, 21, 118–120]. Primary studies would benefit from applying the CFIR “rating rules” which explore the valence (i.e. positive or negative impact) and strength of influence of emergent CFIR constructs [93]. Nevertheless, tabulation of the results facilitated consideration of the relationship between constructs and cross comparison [45].

The inclusion of qualitative, quantitative, and mixed method studies ensured wide exploration of the barriers and facilitators in this context, which meant that different quality assessment tools were applied. Although tools of similar depth were selected, it is notable that the CASP and BGQC tools covered different aspects of quality [26, 32]. For example, the BGQC tool does not explore the relationship between participants and researchers unlike the CASP tool. Therefore, to what extent the quality assessment results are truly comparable between study types is unknown.

To the authors’ knowledge, using a directed content analysis approach when applying the CFIR is novel and helped assess its applicability to the community pharmacy setting. The CFIR constructs captured most emergent data except for two instances within the CFIR outer setting domain. Patients’ perceptions, awareness, and engagement were not encompassed within the “Patient Needs and Resources” construct as the construct focuses on the ability of an organisation to identify and prioritise patient’s needs (which has been criticised elsewhere [121]). The “Cosmopolitanism” construct overlooks the impact of external healthcare professional’s engagement as it centres on networking with external organisations, and further exploration of how cosmopolitanism may be influenced by the infrastructure of healthcare systems is required. Both of these factors would not appear relevant in any other CFIR domain or construct, and we suggest widening the scope of these CFIR constructs accordingly.

Limiting the search to the English language compromised the ability to identify implementation facilitators and barriers internationally. However, 41% (*n* = 16) of included studies were from non-English-speaking nations. During full-text screening, 35 studies could not be accessed. As only 8% (*n* = 29) of the 358 studies screened were included, we estimate only three of these would be eligible for inclusion. Although the study selection process underwent independent validation, alignment of the results to the CFIR was not conducted independently. However, a dedicated CFIR codebook and technical assistance guide was routinely accessed to ensure appropriate alignment to CFIR constructs [93]. Lower quality studies were retained to allow for broad data capture, and removal of the lower quality studies did not affect the representation of the most commonly cited CFIR constructs. Conference abstracts obtained the lowest quality scores—likely to be reflective of their reporting depth—although retaining these ensured representation of the latest research [120].

## Conclusions

Pharmacy staff’s perceptions of the barriers and facilitators to the implementation of national innovations within the community pharmacy setting have been identified. Commonly reported factors which influence implementation include insufficient resources, the views of patients and other healthcare professionals, pharmacy staff perceptions and acceptance of innovations, and belief that innovations were beneficial. Key findings led to the development of a preliminary theory where it is proposed that successful national implementation of community pharmacy innovations requires innovations which are easy to operate, alongside positively engaged patients, pharmacy staff, and other healthcare professionals such as GPs. Potential applications of the results include better directed evaluations and the development of implementation strategies which overcome common barriers and exploit known facilitators. Key recommendations include (1) more robust piloting and sufficient pre-implementation exploration, (2) phased implementation, (3) promotion of whole-team involvement, and (4) more thorough engagement strategies.

## Additional files

Additional file 1: Medline search strategy. (DOCX 27 kb)

Additional file 2: Consolidated Framework for Implementation Research construct descriptions. (DOCX 21 kb)

Additional file 3: Characteristics of included studies. (DOCX 69 kb)

Additional file 4: Full quality assessment results. (DOCX 83 kb)

Additional file 5: Full presentation of constructs and subconstructs. (DOCX 536 kb)

Additional file 6: CFIR constructs represented within the different reviews. (DOCX 33 kb)

## Abbreviations

BGQC: Boynton and Greenhalgh Quality Checklist
CASP: Critical Appraisal Skills Programme
CFIR: Consolidated Framework for Implementation Research
CINAHL: Cumulative Index of Nursing and Allied Health Literature
MADRAC: Malaysian spontaneous adverse drug reporting system
MMAT: Mixed Methods Appraisal Tool
PRISMA: Preferred Reporting Items for Systematic Reviews and Meta-Analysis
