# Supplementary material for: Factors influencing national implementation of innovations within community pharmacy: a systematic review applying the Consolidated Framework for Implementation Research
Source: Implement Sci. 2019 Mar 4;14:21. doi: 10.1186/s13012-019-0867-5 (PMC6398232; doi:10.1186/s13012-019-0867-5)
Supplement: Supplementary file 1 — Medline search strategy. (DOCX 27 kb) [file 13012_2019_867_MOESM1_ESM.docx]

## Additional file 1: Medline search strategy.

| # | **Searches** | **Results** | **Search Type** |
| --- | --- | --- | --- |
| 1 | *Pharmacies/ | 2839 | Advanced |
| 2 | *Community Pharmacy Services/ | 2478 | Advanced |
| 3 | *Pharmacists/ | 7827 | Advanced |
| 4 | *Pharmacists' Aides/ | 381 | Advanced |
| 5 | *Students, Pharmacy/ | 1264 | Advanced |
| 6 | community pharmac*.mp. | 4751 | Advanced |
| 7 | drug store*.mp. | 241 | Advanced |
| 8 | retail pharmac*.mp. [mp=title, abstract, original title, name of substance word, subject heading word, keyword heading word, protocol supplementary concept word, rare disease supplementary concept word, unique identifier] | 367 | Advanced |
| 9 | druggist.mp. | 41 | Advanced |
| 10 | chemist.mp. | 1134 | Advanced |
| 11 | apothecary.mp. | 278 | Advanced |
| 12 | dispensar*.mp. [mp=title, abstract, original title, name of substance word, subject heading word, keyword heading word, protocol supplementary concept word, rare disease supplementary concept word, unique identifier] | 4230 | Advanced |
| 13 | pharmacy technician*.mp. | 433 | Advanced |
| 14 | checking technician*.mp. | 2 | Advanced |
| 15 | student pharmacist*.mp. | 156 | Advanced |
| 16 | pre-registration pharmacist*.mp. | 4 | Advanced |
| 17 | trainee pharmacist*.mp. | 2 | Advanced |
| 18 | medicine counter assistant*.mp. | 4 | Advanced |
| 19 | over the counter assistant*.mp. | 0 | Advanced |
| 20 | medication assistant*.mp. | 7 | Advanced |
| 21 | dispenser*.mp. | 1411 | Advanced |
| 22 | dispensing assistant*.mp. | 0 | Advanced |
| 23 | pharmacy team*.mp. | 59 | Advanced |
| 24 | pharmacy staff*.mp. | 504 | Advanced |
| 25 | 1 or 2 or 3 or 4 or 5 or 6 or 7 or 8 or 9 or 10 or 11 or 12 or 13 or 14 or 15 or 16 or 17 or 18 or 19 or 20 or 21 or 22 or 23 or 24 | 22237 | Advanced |
| 26 | *Health Plan Implementation/ | 1890 | Advanced |
| 27 | *Information Dissemination/ | 6130 | Advanced |
| 28 | *Health Care Reform/ | 20992 | Advanced |
| 29 | *"Diffusion of Innovation"/ | 7439 | Advanced |
| 30 | *Health Planning Technical Assistance/ | 124 | Advanced |
| 31 | *Regional Health Planning/ | 3095 | Advanced |
| 32 | *Education, Pharmacy/ | 3342 | Advanced |
| 33 | *Quality Improvement/ | 5280 | Advanced |
| 34 | Community Health Planning/ | 4662 | Advanced |
| 35 | *Health Systems Plans/ | 76 | Advanced |
| 36 | *Program Development/ | 6517 | Advanced |
| 37 | *Technology, Pharmaceutical/ | 7020 | Advanced |
| 38 | *National Health Programs/ | 18072 | Advanced |
| 39 | *Patient Care/ | 4282 | Advanced |
| 40 | *Patient Care Bundles/ | 87 | Advanced |
| 41 | *Patient Education as Topic/ | 33301 | Advanced |
| 42 | *Patient Education Handout/ | 0 | Advanced |
| 43 | *Health Education/ | 31094 | Advanced |
| 44 | *Evidence-Based Practice/ | 2707 | Advanced |
| 45 | *Practice Guidelines as Topic/ | 30743 | Advanced |
| 46 | *Clinical Protocols/ | 4839 | Advanced |
| 47 | *Patient Selection/ | 14363 | Advanced |
| 48 | *Teach-Back Communication/ | 6 | Advanced |
| 49 | *Consumer Health Information/ | 1624 | Advanced |
| 50 | *Health Promotion/ | 38211 | Advanced |
| 51 | *Patient Safety/ | 4427 | Advanced |
| 52 | intervention*.mp. | 610588 | Advanced |
| 53 | disseminat*.mp. | 102472 | Advanced |
| 54 | implement*.mp. | 253333 | Advanced |
| 55 | adopt*.mp. | 149241 | Advanced |
| 56 | roll* out.mp. | 1040 | Advanced |
| 57 | scale* up.mp. | 6947 | Advanced |
| 58 | knowledge transfer.mp. | 749 | Advanced |
| 59 | uptake*.mp. | 285736 | Advanced |
| 60 | 26 or 27 or 28 or 29 or 30 or 31 or 32 or 33 or 34 or 35 or 36 or 37 or 38 or 39 or 40 or 41 or 42 or 43 or 44 or 45 or 46 or 47 or 48 or 49 or 50 or 51 or 52 or 53 or 54 or 55 or 56 or 57 or 58 or 59 | 1502435 | Advanced |
| 61 | *Health Services Research/ | 13393 | Advanced |
| 62 | *Quality Assurance, Health Care/ | 29565 | Advanced |
| 63 | *"Process Assessment (Health Care)"/ | 1640 | Advanced |
| 64 | *Program Evaluation/ | 8067 | Advanced |
| 65 | *Intervention Studies/ | 324 | Advanced |
| 66 | *Data Collection/ | 12120 | Advanced |
| 67 | *Evaluation Studies as Topic/ | 6256 | Advanced |
| 68 | *Evaluation Studies/ | 0 | Advanced |
| 69 | *Feasibility Studies/ | 127 | Advanced |
| 70 | *Multicenter Studies as Topic/ | 1776 | Advanced |
| 71 | *Pilot Projects/ | 373 | Advanced |
| 72 | *Sampling Studies/ | 815 | Advanced |
| 73 | *"Attitude of Health Personnel"/ | 50003 | Advanced |
| 74 | *Clinical Competence/ | 34208 | Advanced |
| 75 | *Professional Competence/ | 9984 | Advanced |
| 76 | *Health Knowledge, Attitudes, Practice/ | 43019 | Advanced |
| 77 | implementation evaluat*.mp. [mp=title, abstract, original title, name of substance word, subject heading word, keyword heading word, protocol supplementary concept word, rare disease supplementary concept word, unique identifier] | 218 | Advanced |
| 78 | program* evaluat*.mp. | 53623 | Advanced |
| 79 | barrier*.mp. | 175678 | Advanced |
| 80 | enabler*.mp. | 885 | Advanced |
| 81 | facilitator*.mp. | 13629 | Advanced |
| 82 | obstacle*.mp. | 29102 | Advanced |
| 83 | challenge*.mp. | 369073 | Advanced |
| 84 | 61 or 62 or 63 or 64 or 65 or 66 or 67 or 68 or 69 or 70 or 71 or 72 or 73 or 74 or 75 or 76 or 77 or 78 or 79 or 80 or 81 or 82 or 83 | 786381 | Advanced |
| **85** | **25 and 60 and 84** | **1472** | **Advanced** |
| **86** | **limit to English language** | **1417** | **Advanced** |
