# Supplementary material for: Factors influencing national implementation of innovations within community pharmacy: a systematic review applying the Consolidated Framework for Implementation Research
Source: Implement Sci. 2019 Mar 4;14:21. doi: 10.1186/s13012-019-0867-5 (PMC6398232; doi:10.1186/s13012-019-0867-5)
Supplement: Supplementary file 3 — Characteristics of included studies. (DOCX 69 kb) [file 13012_2019_867_MOESM3_ESM.docx]

Additional file 3: Characteristics of included studies.

| **AUTHOR, YEAR, COUNTRY** | **QUALITY ASSESSMENT RESULT** | **AIM** | **INNOVATION DETAILS** | **EVALUATION METHOD** | **SAMPLE PARTICIPANTS** | **RESPONSE** |
| --- | --- | --- | --- | --- | --- | --- |
| **Legislative Change** | | | | | | |
| Allenet et al, 2003, France [1]* | 38% | To describe the opinion and behaviour of pharmacists towards generic substitution. | New law passed permitting substitution between brand and generic drugs based on a list developed by the French Drug Administration alongside a new reimbursement model. | Postal questionnaire | Pharmacy owners sampled through a national pharmacy magazine. | N=1000 questionnaires randomly selected for analysis |
| Chee Ping et al, 2010, Australia [2]* | 79% | To evaluate the impact of reforms of a pharmaceutical benefits scheme on pharmacist perceptions and practices regarding generic medicines. | Generic substitution policy reform which saw the development of a "brand innovation" and generic medicine formularies in, pricing reforms, including a fee of AUD $ 1.50 when a generic medicine was dispensed. | Online questionnaire | Pharmacists including proprietors, managers, employees and locums sampled, via direct mailing and link to questionnaire posted within newsletters. | N=157 |
| Guald et al, 2010, New Zealand [3]* | 63% | To elucidate how non-prescription supply of oseltamivir worked in practice and whether improvements were necessary. | Reclassification of oseltamivir (Tamiflu) for it to be available off prescription under set criteria (e.g. patients over the age of 12 presenting in person during influenza season). | Semi-structured interviews | Pharmacists from a purposive sample of 903 community pharmacies | N=26 |
| Gröber-grätz et al, 2010, Germany [4]* | 50% | To investigate the implementation of Drug Discount Contracts and their impact. | A reformed health insurance act which aimed to reduce expenses saw the entitlement of health insurance companies to make Drug Discount Contracts with pharmaceutical manufactures. If patients had prescriptions for a brand name drug from a different manufacturer than the contract partner, pharmacist are not to supply the prescribed drug but the contract partners. Substitution rules include identical active ingredient, dosage, pack size, indication, and same or exchangeable galenics. | Postal questionnaire | Pharmacy staff (pharmacists, pharmacy assistants, pharmaceutical sales assistants and “others”) sampled through a pharmacy journal. | N=804 |
| Hamrosi et al, 2014, Australia [5]* | 70% | To identify the barriers and facilitators to the utilisation of Consumer Medicine Information. | Standardized Consumer Medicine Information was introduced within Australia, which is brand specific written information for patients about medicines developed by pharmaceutical manufacturers. Guidelines were developed regarding the provision for doctors and pharmacists. | Postal questionnaire | Stratified random national sample (of metropolitan and rural settings) of 1100 pharmacists in New South Wales *NB. GPs were also sampled but this data was not extracted.* | N=349 (34%) |
| Hansford et al, 2007, Great Britain [6]* | 64% | To describe community pharmacists’ views, attitudes and early experiences of over the counter simvastatin. | Simvastatin 10mg was reclassified from a prescription only medication to pharmacy status, making it available for over the counter supply. Licensing restricts sales to those with moderate 10 year risk of a first coronary event. A cardiovascular risk assessment should be conducted by pharmacists. | Postal questionnaire | The main pharmacist with most responsibility for over the counter supply of medicines from a random sample of 2000 pharmacies. | N=1156 (58%) |
| Paudyal et al, 2012, Scotland (UK) [7]* | 64% | To understand pharmacists’ perceived integration into practice, and attitudes to over the counter simvastatin 5 years post reclassification. | See above (Hansford et al) | Postal questionnaire | The main pharmacist with most responsibility for over the counter supply of medicines from all community pharmacies in Scotland (N=1138) | N = 563 (50%) |
| Lonergan et al, 2012, Ireland (UK) [8]^ | 41% | To explore the opinions and experiences of pharmacy staff and patients to guidelines controlling the sale and supply of non-prescription codeine medications in Ireland. | Guidelines developed for pharmacists and retail pharmacy businesses on the safe supply of non-prescription codeine products were developed which restricts supply to improve patient safety. | Semi-structured interviews | Purposive sampling of pharmacy staff (pharmacists, pre-registration pharmacists and pharmacy assistants) from a range of pharmacy types and geographical location.  *NB. Patients were also sampled but this data was not extracted.* | N=10 |
| Weidmann et al, 2011, Great Britain [9]* | 64% | To investigate the experiences, views and attitudes of community pharmacists towards the sale of orlistat. | Orlistat was approved for over the counter sale as a weight loss medication for those over 18 years old, with a BMI of >28kg/m2. Good practice guidelines issued recommends which included BMI testing and the use of additional multivitamin supplements. | Postal questionnaire | Pharmacists from a random selection of 13200 community pharmacies | N=4026 (32.4%) |
| Thomas et al, 2009, England (UK) [10]^ | 25% | To evaluate community pharmacists’ views and understanding of amendments to the controlled drug’s regulations and the challenges faced, and to explore community pharmacists’ perception of the regulations in relation to the disposal of patient-returned controlled drugs. | Amendments to controlled drugs regulations. | Postal questionnaire and semi-structured interviews | Pharmacists from 120 pharmacies in three regions were sampled for questionnaire.  For the qualitative part, pharmacists from 77 community pharmacies in the two primary care trusts were sampled. | N= 63 (32%) for the questionnaires  N=10 for the interviews |
| **Clinical Innovations** | | | | | | |
| Donovan et al, 2016, England (UK) [11]* | 70% | To explore the views, attitudes and perceptions of pharmacy support staff on the Health Living Pharmacy initiative. | The concept of Healthy Living Pharmacies was developed, which were too support the health and wellbeing of patients to improve health outcomes through provision of public health services. Features included a criteria for quality and performance, and having a trained healthy living champion in each pharmacy. Advice on health issues are offered to patients (e.g. smoking cessation and physical activity), services included emergency hormonal contraception and needle exchange schemes. | Semi-structured interviews | Up to three support staff (medicine counter assistants, dispensing assistants, pharmacy technicians, accuracy checking technicians) from accredited Health Living Pharmacies located in the Northumberland region. | N=21 |
| Brooks et al,  2013, England (UK) [12]^ | 47% | To explore the perspectives and experiences of community pharmacy staff who provide public health services on becoming a “Healthy Living Pharmacy”. | See above (Donovan et al) | In-depth interviews | Pharmacy staff (pharmacists, Healthy Living Champions and technicians) located in the Staffordshire region. | N=18 |
| Firth et al , 2015, England (UK) [13]* | 63% | To explore the barriers to the implementation and progression of the Health Living Pharmacy framework. | See above (Donovan et al) | Structured interviews | Pharmacists and Healthy Living Champions working in Healthy Living Pharmacies purposively sampled based on varying deprivation classifications and plans for the pharmacy to progress to next tiers of the Heathy Living Pharmacy framework. | N=22 (n=11 pharmacists, n=11 healthy living champions) |
| Rutter et al, 2015, England (UK) [14]* | 68% | To understand the Healthy Living Champions’ perspective of their role and explore the barriers and facilitators to their performance | See above (Donovan et al) | Semi-structured interviews | Healthy Living Champions sampled in the region of Dudley (n=29). | N=14 (48%) |
| Shevket et al, 2015, England (UK) [15]^ | 35% | To explore Healthy Living Pharmacy staff perspectives on their pharmacy being a Healthy Living Pharmacy and whether this changed over the course of a year | Health and wellbeing service developed within Community pharmacy | In-depth interviews | Purposive sampling of pharmacy staff (pharmacists, healthy living champions and technicians) from Healthy Living Pharmacies from a range of different pharmacy types (i.e. independents/chains) in the Staffordshire region . | N=18, 9 of which had follow up interviews |
| Latif et al, 2016, England (UK) [16]* | 62% | To investigate the “New Medicines Service” implementation process and how it is translating and transformation in practice. | The New Medicines Service was introduced as an advanced service within the community pharmacy contractual framework. This service offers support to improve patient’s adherence of new medicines for specified long term conditions. Patients are invited to the service if they present with a prescription for a new medicine for a long-term condition, and can self-refer, be referred by a GP or nurse or pharmacists initiate the service. There are two patient-pharmacist consultations (either face to-face or via telephone). Guidance questions were provided to the pharmacists to facilitate the patient discussion. | Observations and semi-structured interviews (including short "exit" interviews and full length interviews) | Purposive sample of community pharmacists providing the New Medicine Service who were recruited in the region of East-Midland, South Yorkshire and London, encompassing different ownership types, geographic areas and social deprivation.  *NB. GPs were also sampled but this data was not extracted.* | Observations in 23 community pharmacies  N=27 full length interviews  N=20 shorter "exit" interviews |
| Corlett et al, 2013, England (UK) [17]^ | 33% | To explore community pharmacists’ views and experiences of providing the “New Medicines Service”. | See above (Latif et al) | Focus groups | Convenience sample of pharmacists (locums and managers) in the Kent region. | N=9 (from two focus groups conducted) |
| Lucas et al, 2015, England (UK) [18]* | 71% | To explore community pharmacists’ experiences and perceptions of the “New Medicines Service”. | See above (Latif et al) | Semi-structured interviews | 20 community pharmacists from an area in West Yorkshire (with 123 community pharmacies), purposively chosen to have a range of deprivation categories and pharmacy sites and have provided at least one New Medicine Service. | N=14 (70%) |
| Kaae et al, 2010, Denmark [19]* | 66% | To describe the implementation of the “Inhaler Technique Assessment Service” and factors influencing the sustainability of the service. | The Inhaler Technique Assessment Service included a demonstration of corrected inhaler use for patients with asthma and chronic obstructive pulmonary disease, with a manual describing the technical aspects of inhalation. It was intended for newly diagnosed and existing patients when they hand in an inhaler prescription. Documentation was mandatory, and monthly reports are sent to the Danish Medicines Agency. It takes approximately 10 minutes, with a reimbursement fee of 9 dollars. | Observations, semi-structured interviews, and collection and review of documentary material | Purposive sampling of pharmacies based on stable, increasing or declining provision of the Inhaler Technique Assessment Service. | N=7 pharmacies involved  N= 29 interviews (n=7 pharmacy owners, n=5 pharmacists, n=17 pharmacy assistants) |
| Kaae et al, 2011, Denmark [20]* | 65% | To investigate how organisational factors, particularly leadership style, influence sustainability of the Inhaler Technique Assessment Service | See above (Kaae et al) | Observations, interviews, and collation of written materials that illustrated the implementation process. | Purposive sampling of pharmacy staff from pharmacies of with different geographical range, varying achievements of sustainability and where the pharmacy owner owned the pharmacy during the entire period since the Inhaler Technique Assessment Service launch. | N=4 pharmacies, unclear how many interviewed. |
| Latif et al, 2008, UK [21]* | 55% | To explore factors that affect the number of “Medicine Use Reviews” and investigate attitudes towards its implementation and value. | Medicine Use Reviews are services which involved a consultation to establish patient understanding of their medications. A report was generated and provided to the patient and to their GP, if necessary. Pharmacies can opt in to deliver this service if they meet accreditation requirements. | Postal questionnaire | Convenience sample of 280 accredited pharmacists within one pharmacy chain. | N=167 (60%) |
| Latif et al, 2010, UK [22]^ | 37% | To compare views of “Medicine Use Reviews” to previous results. | See above (Latif et al) | Questionnaire | Pharmacists sample from 300 accredited pharmacies in one pharmacy chain. | N=189 (From 146 pharmacies, 49%) |
| Wilcock et al, 2008, England (UK) [23]* | 42% | To explored perceptions of community pharmacists’ on “Medicines Use Reviews” and its impact on patients. | See above (Latif et al) | Interviews | A purposive sample of 10 community pharmacists from pharmacies providing Medicines Use Reviews, selected based on rurality and if multiple or independent pharmacies.  *NB. GPs were also sampled but this data was not extracted.* | N=10 |
| Lee, 2008, New Zealand [24]* | 48% | To identify where “Medicines Use Review” services are provided by pharmacist and explore the processes and pharmacists' perceptions of the service. | See above (Latif et al) | Postal questionnaire | A sample of all Medicines Use Review accredited pharmacists in New Zealand who had contact details available (n=68) | N=54 (79%) |
| Blenkinsopp et al, 2007, England and Wales (UK) [25]^ | 19% | To investigated community pharmacists’ experience of providing the “Medicines Use Review” and prescription intervention service and the future plans of those not currently providing it. | See above (Latif et al) | Postal questionnaire and focus groups. | Pharmacists from a random stratified 10% sample from 31 regions in England and Wales. | N=767 (71%)  Purposefully selected pharmacists participates in four focus groups in case study primary care organisations.  N=25 |
| Bell et al, 2012, Cambodia [26]* | 65% | To investigate the attitudes and practices of pharmacy-initiated tuberculosis referral service | Pharmacy-based assessment of people with tuberculosis symptoms and referral to treatment centres, including provision of information, counselling and referral documentation. Pharmacies register with the Municipal Health Department to join the programme. | Focus groups | Purposive sampling of pharmacist owners, pharmacists and assistants based on years of experience providing referral services. | N=54 (71%) |
| Hodson et al, 2014, Wales (UK) [27]^ | 29% | To capture views on the “Wales Discharge Medicines” review service. | A Discharge Medicine Review service was introduced to improve the management of medication post-discharge from a care setting. | Online questionnaire | Pharmacists sampled from all community pharmacies in Wales (n=704) | N=143 (20%) |
| Kansana-hoa et al, 2005, Finland [28]* | 65% | To assess implementation of the “TIPPA” patient counselling project. | The TIPPA project promoted patient counselling in community pharmacies. It included an electronic database on medication to support the verbal counselling, a manual on good practice counselling, a website with access to 1600 website links to medical and drug information in three language, a handbook of guidelines for OTC medication, and a handbook of communication skills. | Postal questionnaire | 734 pharmacists randomly sampled from two registers representing 90% of all Finnish pharmacists. | N=376 (51%) |
| Loo et al, 2011, England (UK) [29]^ | 31% | To derive information concerning community pharmacists’ activities and attitudes towards the “National Health Service Health Check”. | The National Health Service Health Check involved risk assessment (e.g. lifestyle assessment and measurement of blood pressure and cholesterol) and risk management (e.g. offering advice on weight management, alcohol consumption) of cardiovascular disease. | Postal questionnaire | Purposive sampling of pharmacists working in pharmacies in areas with higher deprivation and earlier deaths in heart disease and strokes than national standards (N=1301). | N=442 (34%) |
| Paudyal et al, 2010, Scotland (UK) [30]* | 68 % | To obtain pharmacists’ views on the implementation of the “Minor Ailment Service” in Scotland, with objectives to determine the level of service delivery and the barriers and facilitators related to its implementation. | The Minor Ailment Service was initiated as part of the core contract to promote the public to utilize community pharmacies instead of GPs. It involves the provision of free advice for minor ailments, product supply and referral if appropriate for patient’s exempt for prescription payments. It is supported by a national IT network which facilitates remuneration. | Postal questionnaire | Pharmacists with main responsibly in non-prescription medicine supply sampled from all community pharmacies in Scotland excluding those involved in qualitative phase of this evaluation (N=1138). | N=563 (50%) |
| Chaar et al, 2013, Australia [31]* | 74% | To investigate factors affecting provision of opioid substitution treatment in New South Wales pharmacies, with objectives to explore motivators for provision, and factors influencing the success of the provision. | The Australian government developed an opioid substitution treatment programme, involving regular dosing of long action opioid substitutes under supervision free of charge except with a small dispensing fee to pay. Pharmacists can opt-in to deliver this service. | Semi-structured interviews | Purposive selection of owners and pharmacists based on location and type of business setting. | N=35 |
| **Pharmacovigilance** | | | | | | |
| Irujo et al, 2007, Spain [32]* | 57% | To identify the factors that influence community pharmacists’ adverse drug reaction reporting. | As part of a WHO programme for international drug monitoring, community pharmacies participated in reporting adverse drug reactions through spontaneous reporting according to pharmacovigilance regulations developed. | Questionnaire administered in person | Sample of community pharmacists working in the 546 pharmacies in Navarra. | N=78 |
| Bawazir, 2006, Saudi Arabia [33]* | 74% | To assess attitude and behaviour of private community pharmacists towards adverse drug reaction reporting | The Ministry of Health developed a program to early detect unexpected and serious adverse drug reactions, detect increase in frequency of known adverse drug reactions, quality defects of registered products and to publish and disseminate reports. For community pharmacies, adverse drug reaction reporting forms were distributed, databases were developed for recording and storing reports, and an advisory committee established. | Questionnaire administered in person | Stratified random sample of 25% (n=24) of Riyadh private community pharmacies. | N=172 (72%) |
| Duarte et al, 2015, Portugal [34]* | 76% | Evaluate the habits of spontaneous reporting of adverse drug reactions by community pharmacists, their knowledge of the new legislation, and reasons behind potential issues. | The National Pharmacovigilance System has been established since 1992 to allow spontaneous reporting of adverse drug reactions. New legislation was introduced in 2010 which included new definition of what an adverse drug reaction is, the provision of risk management data, and inclusion of patients as reporters. | Open-ended telephone or email questionnaire | Community pharmacists working within the 301 community pharmacies in Southern Portugal. | N=154 (57% of the 271 contactable pharmacies) |
| Elkalmi et al, 2011, Malaysia [35]* | 62% | Evaluate community pharmacists’ attitudes and perceptions to the Malaysian adverse drug reaction programme and determine awareness, involvement, reasons for under-reporting, and what might encourage more ADR reporting. | The adverse drug reporting system involved voluntary reporting, submitted electronically or by post or fax. Both healthcare professionals and patients can report adverse reactions either directly to the Malaysian Spontaneous Adverse Drug Reporting System (MADRAC) or the company marketing the product. | Semi-structured interviews | Purposive and snowball sampling of community pharmacists (proprietors, managers and employee pharmacists) in Penang Island. | N=16 |
| Elkalmi et al, 2014, Malaysia [36]* | 78% | To examine the attitudes, perception and barriers to adverse drug reactions reporting. | See above (Elkalmi et al) | Postal questionnaire | 470 pharmacists (proprietors, managers, employee and “other” pharmacists) practicing in four northern Malaysian states. | N=116 (25%) |
| Van Grootheest et al, 2002, The Netherlands [37]* | 62% | To gain insight into the attitudes and reporting behaviour of community pharmacist in the Netherlands to adverse drug reactions reporting. | The reporting of suspected adverse drug reaction is a WHO programme for international drug monitoring. Reports are collected and analysed by the Netherlands Pharmacovigilance Centre. Pharmacist reporting of adverse drug reactions is not compulsory. | Postal questionnaire | Stratified random sample of pharmacists (owner, managers, and second pharmacists) from 200 community pharmacies. | N=147 (74%) |
| **e-Technology** | | | | | | |
| Rahimi et al, 2011, Sweden [38]* | 50% | To examine the introduction of an ePrescribing into the practice of pharmacy. | The Swedish IEPS (Integrated electronic prescribing system) was developed which allowed prescriptions generated in wards or physician offices to be transmitted electronically to pharmacies. ePrescriptions are transmitted electronically and stored online with patient and pharmacy access. The dispensing process is preformed from the computer screen.  Patients can choose any pharmacy to collect their medication, and the new prescriptions were retrieved by entering the patient’s social security number. | Postal questionnaire | All pharmacists (N=74) in the Linkoping region. | N=52 (70%) |
| Hammar et al, 2010, Sweden [39]* | 61% | To evaluate Swedish pharmacists’ attitudes towards ePrescribing. | See above (Rahimi et al) | Online questionnaire | Random sample of 500 pharmacists employed within the approximated 900 community pharmacies which handles prescription medicines and had an employee e-mail address (4674 pharmacists met inclusion criteria). | N=259 (52%) |

**References**

1. Allenet B, Barry H. Opinion and behaviour of pharmacists towards the substitution of branded drugs by generic drugs: survey of 1,000 French community pharmacists. Pharm World Sci. 2003;25:197-202.

2. Ping CC, March G, Clark A, Gilbert A, Hassali MA, Bahari MB. A Web-Based Survey on Australian Community Pharmacists' Perceptions and Practices of Generic Substitution. J Generic Med. 2010;7:342-53. doi: doi:10.1057/jgm.2010.23.

3. Gauld N, Kelly F, Shaw J. Is non-prescription oseltamivir availability under strict criteria workable? A qualitative study in New Zealand. J Antimicrob Chemother. 2011;66:201-4. doi: 10.1093/jac/dkq409.

4. Gröber-Grätz D, Gulich M. Impact of drug discount contracts on pharmacies and on patients’ drug supply. J Public Health. 2010;18:583-9. doi: 10.1007/s10389-010-0338-6.

5. Hamrosi KK, Raynor DK, Aslani P. Enhancing provision of written medicine information in Australia: pharmacist, general practitioner and consumer perceptions of the barriers and facilitators. BMC Health Serv Res. 2014;14:183. doi: 10.1186/1472-6963-14-183.

6. Hansford D, Cunningham S, John D, McCaig D, Stewart D. Community pharmacists’ views, attitudes and early experiences of over-the-counter simvastatin. Pharm World Sci. 2007;29:380-5. doi: 10.1007/s11096-007-9084-4.

7. Paudyal V, Hansford D, Cunningham S, Stewart D. Pharmacists' perceived integration into practice of over-the-counter simvastatin five years post reclassification. Int J Clin Pharm. 2012;34:733-8. doi: 10.1007/s11096-012-9668-5.

8. Lonergan C, O'Grady M, Byrne S. An exploratory study of codeine sales restrictions within Irish pharmacies, a qualitative study. Int J Pharm Pract. 2012;20:43.

9. Weidmann AE, Cunningham S, Gray G, Hansford D, McLay J, Broom J, et al. Over-the-counter orlistat: early experiences, views and attitudes of community pharmacists in Great Britain. Int J Clin Pharm. 2011;33:627-33. doi: 10.1007/s11096-011-9516-z.

10. Thomas T, Hathiari A, Benson C, Oladosu B. Controlled drugs: are they being controlled? An evaluation of the views, understanding and implementation of the amended Controlled Drug Regulations (2006) by community pharmacists. Int J Pharm Pract. 2009;17:75.

11. Donovan GR, Paudyal V. England's Healthy Living Pharmacy (HLP) initiative: Facilitating the engagement of pharmacy support staff in public health. Res Social Adm Pharm. 2016;12:281-92. doi: 10.1016/j.sapharm.2015.05.010.

12. Brooks D, Hopp A, White S. Perspectives of community pharmacy staff on Healthy Living Pharmacies: A qualitative study. Int J Clin Pharm. 2013;21:16.

13. Firth H, Todd A, Bambra C. Benefits and barriers to the public health pharmacy: a qualitative exploration of providers’ and commissioners’ perceptions of the Healthy Living Pharmacy Framework. Perspect Public Health. 2015;135:251-6.

14. Rutter P, Vryaparj G. Qualitative exploration of the views of healthy living champions from pharmacies in England. Int J Clin Pharm. 2015;37:27-30. doi: 10.1007/s11096-014-0055-2.

15. Shevket O, White S. A qualitative follow-up study of the perspectives of community pharmacy staff on Healthy Living Pharmacies. Int J Clin Pharm. 2015;23:69-70.

16. Latif A, Waring J, Watmough D, Barber N, Chuter A, Davies J, et al. Examination of England's New Medicine Service (NMS) of complex health care interventions in community pharmacy. Res Social Adm Pharm. 2016;12:966-89. doi: 10.1016/j.sapharm.2015.12.007.

17. Corlett S, Dodds L. The New Medicines Service; Initial views and early experiences of Community Pharmacists in Kent. Int J Clin Pharm. 2013;21:96-7.

18. Lucas B, Blenkinsopp A. Community pharmacists' experience and perceptions of the New Medicines Service (NMS). Int J Pharm Pract. 2015;23:399-406. doi: 10.1111/ijpp.12180.

19. Kaae S, Sondergaard B, Stig L, Traulsen JM. Sustaining delivery of the first publicly reimbursed cognitive service in Denmark: A cross-case analysis. Int J Pharm Pract. 2010;18:21-7.

20. Kaae S, Søndergaard B, Haugbølle LS, Traulsen JM. The relationship between leadership style and provision of the first Danish publicly reimbursed cognitive pharmaceutical service—A qualitative multicase study. Res Social Adm Pharm. 2011;7:113-21. doi: 10.1016/j.sapharm.2010.03.001.

21. Latif A, Boardman H. Community pharmacists' attitudes towards medicines use reviews and factors affecting the numbers performed. Pharm World Sci. 2008;30:536-43.

22. Latif A, Mahmood K, Boardman H. Medicines Use Reviews – how have pharmacists’ views changed? Int J Clin Pharm. 2010;18:69-70.

23. Wilcock M, Harding G. What do pharmacists think of MURs and do they change prescribed medication? Pharm J. 2007;281:163.

24. Lee E, Braund R, Tordoff J. Examining the first year of Medicines Use Review services provided by pharmacists in New Zealand: 2008. N Z Med J. 2009;122:3566.

25. Blenkinsopp A, Celino G, Bond C, Inch J, Gray N. Community pharmacists’ experience of providing medicines use reviews: findings from the national evaluation of the community pharmacy contractual framework. Int J Clin Pharm. 2007;15:845-6.

26. Bell CA, Eang MT, Dareth M, Rothmony E, Duncan GJ, Saini B. Provider perceptions of pharmacy-initiated tuberculosis referral services in Cambodia, 2005-2010. Int J Tuberc Lung Dis. 2012;16:1086-91.

27. Hodson K, James D, Smith M, Hughes L, Blenkinsopp A, Cohen D, et al. Evaluation of the Discharge Medicines Review Service in Wales: community and hospital pharmacists’ views. Int J Clin Pharm. 2014;22:6.

28. Kansanaho H, Puumalainen I, Varunki M, Ahonen R, Airaksinen M. Implementation of a professional program in Finnish community pharmacies in 2000-2002. Patient Educ Couns. 2005;57:272-9.

29. Loo RL, Diaper C, Salami OT, Kundu M, Lalkia M, Airhiavbere E, et al. The NHS Health Check: The views of community pharmacists. Int J Pharm Pract. 2011;19:13.

30. Paudyal V, Hansford D, Scott Cunningham IT, Stewart D. Cross-sectional survey of community pharmacists' views of the electronic Minor Ailment Service in Scotland. Int J Pharm Pract. 2010;18:194-201. doi: 10.1111/j.2042-7174.2010.00042.x.

31. Chaar BB, Wang H, Day CA, Hanrahan JR, Winstock AR, Fois R. Factors influencing pharmacy services in opioid substitution treatment. Drug Alcohol Rev. 2013;32:426-34.

32. Irujo M, Beitia G, Bes-Rastrollo M, Figueiras A, Hernandez-Diaz S, Lasheras B. Factors that influence under-reporting of suspected adverse drug reactions among community pharmacists in a Spanish region. Drug Saf. 2007;30:1073-82.

33. Bawazir S. ATTITUDE OF COMMUNITY PHARMACISTS IN SAUDI ARABIA TOWARDS ADVERSE DRUG REACTION REPORTING. Saudi Pharm J. 2006;14:75-83.

34. Duarte M, Ferreira P, Soares M, Cavaco A, Martins AP. Community pharmacists’ attitudes towards adverse drug reaction reporting and their knowledge of the new pharmacovigilance legislation in the southern region of Portugal: a mixed methods study. Drugs & Therapy Perspectives. 2015;31:316-22. doi: 10.1007/s40267-015-0227-8.

35. Elkalmi RM, Hassali MA, Ibrahim MIM, Liau SY, Awaisu A. A qualitative study exploring barriers and facilitators for reporting of adverse drug reactions (ADRs) among community pharmacists in Malaysia. J Pharm Health Serv Res. 2011;2:71-8.

36. Elkalmi RM, Hassali MA, Ibrahim MI, Jamshed SQ, Al-Lela OQ. Community pharmacists' attitudes, perceptions, and barriers toward adverse drug reaction reporting in Malaysia: a quantitative insight. J Patient Saf. 2014;10:81-7. doi: 10.1097/pts.0000000000000051.

37. Van Grootheest AC, den Berg LTWdJ-v, Mes K. Attitudes of community pharmacists in the Netherlands towards adverse drug reaction reporting. Int J Pharm Pract. 2002;10:267-72. doi: 10.1211/096176702776868460.

38. Rahimi B, Timpka T. Pharmacists' views on integrated electronic prescribing systems: associations between usefulness, pharmacological safety, and barriers to technology use. Eur J Clin Pharmacol. 2011;67:179-84. doi: 10.1007/s00228-010-0936-9.

39. Hammar T, Nystrom S, Petersson G, Rydberg T, Astrand B. Swedish pharmacists value eprescribing: A survey of a nationwide implementation. J Pharm Health Serv Res. 2010;1:23-32.
