# Supplementary material for: Factors influencing national implementation of innovations within community pharmacy: a systematic review applying the Consolidated Framework for Implementation Research
Source: Implement Sci. 2019 Mar 4;14:21. doi: 10.1186/s13012-019-0867-5 (PMC6398232; doi:10.1186/s13012-019-0867-5)
Supplement: Supplementary file 4 — Full quality assessment results. (DOCX 83 kb) [file 13012_2019_867_MOESM4_ESM.docx]

## Additional file 4: Full quality assessment results.

**Table S1** Quality assessment results of qualitative studies.

|  | **Lonergan 2012*** | **Gauld 2011*** | **Kaae 2010*** | **Bell 2012*** | **Shevket 2015^** | **Firth 2015*** | **Lucas 2015*** | **Brooks 2013^** | **Corlett 2013^** | **Rutter 2015*** | **Kaae 2011*** | **Donovan 2016*** | **Chaar 2013*** | **Latif 2016*** | **Wilcock 2008*** | **Elkami 2011*** |
| --- | --- | --- | --- | --- | --- | --- | --- | --- | --- | --- | --- | --- | --- | --- | --- | --- |
| **IS THERE A CLEAR STATEMENT OF THE AIMS OF THE RESEARCH?** | | | | | | | | | | | | | | | | |
| *What the goal of the research was?* | y | y | y | y | y | y | y | y | y | y | y | y | y | y | y | y |
| *Why it was thought to be important?* | y | y | y | y | y | y | y | y | y | y | y | y | y | y | y | y |
| *It's relevance?* | y | y | y | y | y | y | y | y | y | y | y | y | y | y | y | y |
| **IS A QUALITATIVE METHODOLOGY APPROPRIATE?** | | | | | | | | | | | | | | | | |
| *Does the research seek to interpret or illuminate the actions and/or subjective experiences of research participants?* | y | y | y | y | y | y | y | y | y | y | y | y | y | y | y | y |
| **WAS THE RESEARCH DESIGN APPROPRIATE TO ADDRESS THE AIMS OF THE RESEARCH?** | | | | | | | | | | | | | | | | |
| *Has the researcher justified the research design? (i.e. have they discussed how they decided which method to use?* | n | n | y | n | y | n | y | y | n | n | n | n | y | n | n | y |
| **WAS THE RECRUITMENT STRATEGY APPROPRIATE TO THE AIMS OF THE RESEARCH?** | | | | | | | | | | | | | | | | |
| *Is it explained how the individual participants were selected?* | p | y | y | y | n | y | y | n | n | y | y | y | y | y | y | y |
| *Is it explained why the participants selected were most appropriate to provide access to the type of knowledge sought by the study?* | n | y | y | n | y | n | y | y | n | y | y | y | y | n | n | n |
| *Was there are any discussions around recruitment (e.g. why some people chose not to take part)* | n | n | p | n | n | n | n | n | n | y | p | n | y | n | n | n |
| **WAS THE DATA COLLECTED IN A WAY THAT ADDRESSED THE RESEARCH ISSUE?** | | | | | | | | | | | | | | | | |
| *Was the setting for data collection justified?* | y | y | y | y | n | p | p | p | n | n | y | n | y | y | y | n |
| *Is it clear how data was collected (e.g. focus group, semi-structured interview etc.)?* | y | y | y | y | p | y | y | y | y | y | y | y | y | y | y | y |
| *Did the researcher justify the methods chosen?* | n | n | n | n | n | y | y | n | n | n | y | n | n | p | n | n |
| *Did the researcher make the methods explicit (e.g. for interview method, is there an indication of how interviews were conducted, or did they use a topic guide)?* | n | p | p | y | p | y | y | p | n | p | p | y | y | y | n | p |
| *If methods were modified during the study, has the researcher explained how and why?* | n/a | p | n/a | n/a | n/a | p | n/a | n/a | n/a | n/a | y | n/a | n | p | n/a | n/a |
| *Is the form of data clear (e.g. tape recordings, video material, notes etc)?* | y | y | y | y | y | y | y | y | y | y | y | y | y | y | y | y |
| *Did the researcher discuss saturation of data?* | y | y | n | y | n | y | n | n | n | y | y | y | y | n | n | y |
| **HAS THE RELATIONSHIP BETWEEN RESEARCHER AND PARTICIPANTS BEEN ADEQUATELY CONSIDERED?** | | | | | | | | | | | | | | | | |
| *Did the researcher critically examined their own role, potential bias and influence during:* | | | | | | | | | | | | | | | | |
| *a) Formulation of research question* | n | n | n | n | n | n | n | n | n | n | n | n | n | n | n | n |
| *b) Data collection, including sample recruitment and choice of location* | n | p | n | n | n | n | p | n | n | y | n | y | n | n | n | n |
| *How the researcher responded to events during the study and whether they considered the implications of any changes in the research design?* | n | n | n | n | n | n | n | n | n | n | n | n | n | n | n | n |
| **HAVE ETHICAL ISSUES BEEN TAKEN INTO CONSIDERATION?** | | | | | | | | | | | | | | | | |
| *Is there sufficient details of how the research was explained to participants for the reader to assess whether ethical standards were maintained?* | n | y | n | y | n | y | y | n | n | n | n | y | y | y | n | y |
| *Did the researcher discusses issues raised by the study (e.g. issues around informed consent or confidentiality or how they have handled the effects of the study on the participants during and after the study)* | n | n | y | n | n | n | y | n | n | n | n | n | n | n | n | n |
| *Was approval sought from the ethics committee?* | y | y | n/a | y | y | y | y | y | y | y | n | y | y | y | n | n |
| **WAS THE DATA ANALYSIS SUFFICIENTLY RIGOROUS?** | | | | | | | | | | | | | | | | |
| *Was there is an in-depth description of the analysis process?* | n | y | y | y | n | y | y | n | n | y | y | y | y | y | y | y |
| *If thematic analysis was used, is it clear how the categories/themes were derived from the data?* | n | y | y | y | n | y | y | n | n | y | y | y | y | y | y | y |
| *Did the researcher explain how the data presented were selected from the original sample to demonstrate the analysis process?* | n | n | n | n | n | n | n | n | n | y | n/a | n | y | n | n | y |
| *Was sufficient data presented to support the findings?* | n | n | y | y | n | y | y | y | y | y | p | y | y | y | y | y |
| *Was contradictory data taken into account?* | u | y | y | y | u | y | y | n | n | n | y | y | y | y | n | y |
| *Did the researcher critically examine their own role, potential bias and influence during analysis and selection of data for presentation?* | n | y | n | n | n | n | n | n | n | y | n | n | n | n | n | n |
| **IS THERE A CLEAR STATEMENT OF FINDINGS?** | | | | | | | | | | | | | | | | |
| *Are the findings explicit?* | y | y | y | y | y | y | y | y | y | y | y | y | y | y | p | y |
| *Is there adequate discussion of the evidence both for and against the researchers’ arguments?* | n | y | y | y | n | p | p | p | n | y | y | y | y | p | p | y |
| *Did the researcher discuss the credibility ? (e.g. triangulation, respondent validation, more than one analyst.)* | n | n | n | n | n | n | n | n | n | n | n | n | n | p | n | n |
| *Are the findings discussed in relation to the original research question?* | n | y | y | y | y | y | y | y | y | y | y | y | y | y | y | y |
| **HOW VALUABLE IS THE RESEARCH?** | | | | | | | | | | | | | | | | |
| *Did the researcher discusses the contribution the study makes to existing knowledge or understanding e.g. do they consider the findings in relation to current practice or policy, or relevant research-based literature?* | y | y | y | y | p | y | y | y | n | y | y | y | y | y | y | y |
| *Did they identify new areas where research is necessary?* | y | n | n | y | n | n | n | n | n | n | n | y | y | y | n | n |
| *Did the researchers discuss whether or how the findings can be transferred to other populations or considered other ways the research may be used?* | y | n | y | p | n | y | y | y | y | y | y | y | n | n | n | y |
| **RESULTS** | | | | | | | | | | | | | | | | |
| Applicable Questions (out of 34) | 33 | 34 | 32 | 33 | 33 | 34 | 33 | 33 | 33 | 33 | 33 | 33 | 34 | 34 | 33 | 33 |
| Max Score | 66 | 68 | 64 | 66 | 66 | 68 | 66 | 66 | 66 | 66 | 66 | 66 | 68 | 68 | 66 | 66 |
| Score | 27 | 43 | 42 | 43 | 23 | 43 | 47 | 31 | 22 | 45 | 43 | 46 | 50 | 42 | 28 | 41 |
| Percentage | 40.91 | 63.24 | 65.63 | 65.15 | 34.85 | 63.24 | 71.21 | 46.97 | 33.33 | 68.18 | 65.15 | 69.70 | 73.53 | 61.76 | 42.42 | 62.12 |

*peer-reviewed journal paper, ^conference abstract, y = yes (Score of 2), p = partially (Score of 1) n = no (Score of 0), u = unclear (Score of 0)

**Table S2** Quality assessment of questionnaire design studies.

|  | **Chee Ping 2010*** | | **Gröber-grätz 2010*** | | **Weidmann 2011*** | | **Paudyal 2012*** | **Hamrosi 2014*** | | **Allenet 2003*** | | **Hansford 2007*** | | **Latif 2008*** | **Kansanahoa 2005*** | | | **Loo 2011^** | **Paudyal 2010*** | **Hodson 2014^** | **Latif 2010^** | **Lee 2008*** |
| --- | --- | --- | --- | --- | --- | --- | --- | --- | --- | --- | --- | --- | --- | --- | --- | --- | --- | --- | --- | --- | --- | --- |
| **APPROPRIATE RESEARCH QUESTION AND DESIGN?** | | | | | | | | | | | | | | | | | | | | | | |
| *Was there a clear research question?* | y | | y | | y | | y | y | | y | | y | | y | y | | | y | y | y | y | y |
| *Was this important and sensible?* | y | | y | | y | | y | y | | y | | y | | y | y | | | y | y | y | y | y |
| *Was a questionnaire the most appropriate research design for this question?* | y | | y | | y | | y | y | | y | | y | | y | y | | | y | y | y | y | y |
| **APPROPRIATE SAMPLING?** | | | | | | | | | | | | | | | | | | | | | | |
| *Was the sampling frame sufficiently large?* | y | | y | | y | | y | y | | y | | y | | y | y | | | y | y | y | y | y |
| *Was the sampling frame sufficiently representative?* | y | | n | | y | | y | y | | y | | y | | n | y | | | y | y | y | n | y |
| *Did all participants in the sample understand what was required of them?* | u | | u | | y | | u | y | | u | | y | | u | y | | | u | u | u | u | y |
| *Did all participants in the sample attribute the same meaning to the terms in the questionnaire?* | u | | u | | u | | u | u | | u | | u | | u | u | | | u | u | u | u | u |
| **APPROPRIATE INSTRUMENT?** | | | | | | | | | | | | | | | | | | | | | | |
| *Are there any claims for reliability?* | y | | n | | n | | n | n | | n | | n | | n | n | | | n | n | n | n | n |
| *Are claims for reliability justified?* | y | | n/a | | n/a | | n/a | n/a | | n/a | | n/a | | n/a | n/a | | | n/a | n/a | n/a | n/a | n/a |
| *Are there any claims for validity?* | y | | n | | y | | y | n | | n | | y | | y | n | | | y | y | n | n | n |
| *Are claims for validity justified?* | y | | n/a | | y | | y | n/a | | n/a | | y | | n | n/a | | | p | y | n/a | n/a | n/a |
| *Did the questions cover all relevant aspects of the problem?* | p | | y | | y | | y | y | | y | | u | | n | p | | | u | y | u | u | n |
| *Were questions presented in a non-threatening and non-directive way?* | y | | y | | u | | y | y | | y | | u | | y | y | | | u | y | u | u | u |
| *Were open-ended (qualitative) used appropriately?* | n/a | | n/a | | u | | n/a | n/a | | n/a | | u | | n/a | p | | | u | y | u | n/a | u |
| *Were closed ended (quantitative) questions used appropriately?* | y | | y | | u | | y | y | | y | | y | | y | y | | | u | y | u | u | u |
| *Was a pilot version administer to participants’ representative of those in the sampling frame?* | y | | u | | y | | y | y | | u | | y | | p | n | | | n | y | y | n (24, 30) | y |
| *Following piloting , was the instrument modified accordingly if required if required?* | y | | u | | y | | p | y | | u | | y | | u | n/a | | | n/a | u | u | n/a | u |
| **APPROPRIATE RESPONSE?** | | | | | | | | | | | | | | | | | | | | | | |
| *Was satisfactory response rate achieved?* | u | | u | | n | | n | n | | u | | y | | y | y | | | n | n | n | y | y |
| *Have non-responders been accounted for?* | n/a | | n | | n | | n | n | | n | | n | | n | n | | | n | p | n | n | y |
| **APPROPRIATE CODING AND ANALYSIS?** | | | | | | | | | | | | | | | | | | | | | | |
| *Was the analysis appropriate (e.g. statistical analysis for quantitative answers, qualitative analysis for open-ended questions)?* | y | | y | | y | | y | y | | n | | y | | y | y | | | u | y | u | y | y |
| *Were the correct techniques used?* | y | | y | | y | | y | y | | n | | y | | y | y | | | u | y | u | y | u |
| *Were adequate measures in place to maintain accuracy of data?* | u | | u | | u | | u | u | | u | | u | | u | u | | | u | n | u | u | u |
| **APPROPRIATE PRESENTATION OF RESULTS?** | | | | | | | | | | | | | | | | | | | | | | |
| *Have all relevant results (“significant” and “non-significant”) been reported?* | y | | y | | y | | n | y | | n | | u | | y | y | | | u | p | n | n | u |
| *Was data dredging avoided (i.e. analyses that were not ‘hypothesis driven’)?* | n/a | | n/a | | n/a | | n/a | n/a | | u | | n/a | | n/a | n/a | | | n/a | n/a | n/a | n/a | n/a |
| **RESULTS** | | | | | | | | | | | | | | | | | | | | | | |
| Applicable Questions | 21 | | 20 | | 22 | | 21 | 20 | | 21 | | 22 | | 21 | 20 | | | 21 | 22 | 21 | 19 | 21 |
| Max Score | 42 | | 40 | | 44 | | 42 | 40 | | 42 | | 44 | | 42 | 40 | | | 42 | 44 | 42 | 38 | 42 |
| Score | 33 | | 20 | | 28 | | 27 | 28 | | 16 | | 28 | | 23 | 26 | | | 13 | 30 | 12 | 14 | 20 |
| Percentage | 78.57 | | 50.00 | | 63.64 | | 64.29 | 70.00 | | 38.10 | | 63.64 | | 54.76 | 65.00 | | | 30.95 | 68.18 | 28.57 | 36.84 | 47.62 |
|  | **Duarte 2015*** | **Elkami 2014*** | | **Irujo 2007*** | | **Van Grootheest 2002*** | | | **Bawazir 2006*** | | **Hammar 2010*** | | **Rahimi 2011*** | | | |  |  |  |  |  |  |
| **APPROPRIATE RESEARCH QUESTION AND DESIGN?** | | | | | | | | | | | | | | | |  |  |  |  |  |  |  |
| *Was there a clear research question?* | y | y | | y | | y | | | y | | y | | y | | | |  |  |  |  |  |  |
| *Was this important and sensible?* | y | y | | y | | y | | | y | | y | | y | | | |  |  |  |  |  |  |
| *Was a questionnaire the most appropriate research design for this question?* | y | y | | y | | y | | | y | | y* | | y | | | |  |  |  |  |  |  |
| **APPROPRIATE SAMPLING?** | | | | | | | | | | | | | | | |  |  |  |  |  |  |  |
| *Was the sampling frame sufficiently large?* | y | y | | n | | y | | | y | | y | | n | | | |  |  |  |  |  |  |
| *Was the sampling frame sufficiently representative?* | y | n | | y | | y | | | n | | y | | n | | | |  |  |  |  |  |  |
| *Did all participants in the sample understand what was required of them?* | y | u | | y | | u | | | y | | y | | u | | | |  |  |  |  |  |  |
| *Did all participants in the sample attribute the same meaning to the terms in the questionnaire?* | u | u | | u | | u | | | u | | u | | u | | | |  |  |  |  |  |  |
| **APPROPRIATE INSTRUMENT?** | | | | | | | | | | | | | | | |  |  |  |  |  |  |  |
| *Are there any claims for reliability?* | n/a | y | | n | | n | | | y | | n | | n | | | |  |  |  |  |  |  |
| *Are claims for reliability justified?* | n/a | y | | n/a | | n/a | | | y | | n/a | | n/a | | | |  |  |  |  |  |  |
| *Are there any claims for validity?* | n | y | | n | | n | | | y | | y | | y | | | |  |  |  |  |  |  |
| *Are claims for validity justified?* | n/a | y | | n/a | | n/a | | | u | | u | | y | | | |  |  |  |  |  |  |
| *Did the questions cover all relevant aspects of the problem?* | p | y | | y | | y | | | y | | y* | | n | | | |  |  |  |  |  |  |
| *Were questions presented in a non-threatening and non-directive way?* | y | y | | y | | y | | | y | | y | | y | | | |  |  |  |  |  |  |
| *Were open-ended (qualitative) used appropriately?* | y | n/a | | y | | y | | | y | | y | | n/a | | | |  |  |  |  |  |  |
| *Were closed ended (quantitative) questions used appropriately?* | n/a | y | | y | | y | | | y | | y | | y | | | |  |  |  |  |  |  |
| *Was a pilot version administer to participants’ representative of those in the sampling frame?* | y | y | | u | | u | | | y | | y | | n | | | |  |  |  |  |  |  |
| *Following piloting , was the instrument modified accordingly if required if required?* | y | u | | y | | y | | | u | | u | | n/a | | | |  |  |  |  |  |  |
| **APPROPRIATE RESPONSE?** | | | | | | | | | | | | | | | |  |  |  |  |  |  |  |
| *Was satisfactory response rate achieved?* | y | n | | y | | y | | | y | | y | | y | | | |  |  |  |  |  |  |
| *Have non-responders been accounted for?* | n | n | | n | | n | | | y | | n | | y | | | |  |  |  |  |  |  |
| **APPROPRIATE CODING AND ANALYSIS?** | | | | | | | | | | | | | | | |  |  |  |  |  |  |  |
| *Was the analysis appropriate (e.g. statistical analysis for quantitative answers, qualitative analysis for open-ended questions)?* | y | y | | p | | p | | | p | | p | | y | | | |  |  |  |  |  |  |
| *Were the correct techniques used?* | y | y | | p | | p | | | p | | u | | n | | | |  |  |  |  |  |  |
| *Were adequate measures in place to maintain accuracy of data?* | u | u | | u | | u | | | y | | u | | u | | | |  |  |  |  |  |  |
| **APPROPRIATE PRESENTATION OF RESULTS?** | | | | | | | | | | | | | | | |  |  |  |  |  |  |  |
| *Have all relevant results (“significant” and “non-significant”) been reported?* | y | y | | u | | y | | | n | | p | | n | | | |  |  |  |  |  |  |
| *Was data dredging avoided (i.e. analyses that were not ‘hypothesis driven’)?* | n/a | n/a | | n/a | | n/a | | | n/a | | n/a | | n/a | | | |  |  |  |  |  |  |
| **RESULTS** | | | | | | | | | | | | | | | |  |  |  |  |  |  |  |
| Applicable Questions | 19 | 22 | | 21 | | 21 | | | 23 | | 22 | | 20 | | | |  |  |  |  |  |  |
| Max Score | 38 | 44 | | 42 | | 42 | | | 46 | | 44 | | 40 | | | |  |  |  |  |  |  |
| Score | 29 | 30 | | 24 | | 26 | | | 34 | | 27 | | 20 | | | |  |  |  |  |  |  |
| Percentage | 76.32 | 68.18 | | 57.14 | | 61.9 | | | 73.91 | | 61.36 | | 50.00 | | | |  |  |  |  |  |  |

*peer-reviewed journal paper, ^conference abstract, y = yes (Score of 2), p = partially (Score of 1) n = no (Score of 0), u = unclear (Score of 0)

**Table S3** Quality assessment results of mixed method studies.

|  | **Blenkinsopp 2007^** | **Thomas 2009^** |
| --- | --- | --- |
| **MMAT Screening** | | |
| **GENERAL SCREENING QUESTIONS** | | |
| *Are there clear qualitative and quantitative research questions (or objectives*), or a clear mixed methods question (or objective*)?* | y | y |
| *Do the collected data address the research question (objective)? E.g., consider whether the follow-up period is long enough for the outcome to occur (for longitudinal studies or study components).* | y | y |
| **MIXED METHODS METHODOLOGICAL QUALITY CRITERIA** | | |
| *Is the mixed methods research design relevant to address the qualitative and quantitative research questions (or objectives), or the qualitative and quantitative aspects of the mixed methods question (or objective)? (E.g., the rationale for integrating qualitative and quantitative methods to answer the research question is explained.)* | n | y |
| *Is the integration of qualitative and quantitative data (or results) relevant to address the research question (objective)? (E.g., there is evidence that data gathered by both research methods was brought together to form a complete picture, and answer the research question; authors explain when integration occurred (during the data collection-analysis or/and during the interpretation of qualitative and quantitative results); they explain how integration occurred and who participated in this integration.)* | u | p |
| *Is appropriate consideration given to the limitations associated with this integration, e.g., the divergence of qualitative and quantitative data (or results)?* | n | n |
| **Qualitative Quality Assessment** | | |
| **WAS THE RECRUITMENT STRATEGY APPROPRIATE TO THE AIMS OF THE RESEARCH?** | | |
| *Is it explained how the individual participants were selected?* | y | y |
| *Is it explained why the participants selected were most appropriate to provide access to the type of knowledge sought by the study?* | n | n |
| *Was there are any discussions around recruitment (e.g. why some people chose not to take part)* | n | n |
| **WAS THE DATA COLLECTED IN A WAY THAT ADDRESSED THE RESEARCH ISSUE?** | | |
| *Was the setting for data collection justified?* | y | n |
| *Is it clear how data was collected (e.g. focus group, semi-structured interview etc.)?* | y | y |
| *Did the researcher justify the methods chosen?* | n | n |
| *Did the researcher make the methods explicit (e.g. for interview method, is there an indication of how interviews were conducted, or did they use a topic guide)?* | n | n |
| *If methods were modified during the study, has the researcher explained how and why?* | n | n/a |
| *Is the form of data clear (e.g. tape recordings, video material, notes etc)?* | n | y |
| *Did the researcher discuss saturation of data?* | n | n |
| **HAS THE RELATIONSHIP BETWEEN RESEARCHER AND PARTICIPANTS BEEN ADEQUATELY CONSIDERED?** | | |
| *Did the researcher critically examined their own role, potential bias and influence during:* |  |  |
| *a) Formulation of research question* | n | n |
| *b) Data collection, including sample recruitment and choice of location* | n | n |
| *How the researcher responded to events during the study and whether they considered the implications of any changes in the research design?* | n | n |
| **HAVE ETHICAL ISSUES BEEN TAKEN INTO CONSIDERATION?** | | |
| *Is there sufficient details of how the research was explained to participants for the reader to assess whether ethical standards were maintained?* | n | n |
| *Did the researcher discusses issues raised by the study (e.g. issues around informed consent or confidentiality or how they have handled the effects of the study on the participants during and after the study)* | n | n |
| *Was approval sought from the ethics committee?* | n | n |
| **WAS THE DATA ANALYSIS SUFFICIENTLY RIGOROUS?** | | |
| *Was there is an in-depth description of the analysis process?* | n | n |
| *If thematic analysis was used, is it clear how the categories/themes were derived from the data?* | n/a | n |
| *Did the researcher explain how the data presented were selected from the original sample to demonstrate the analysis process?* | n/a | n |
| *Was sufficient data presented to support the findings?* | n | y |
| *Was contradictory data taken into account?* | n | n |
| *Did the researcher critically examine their own role, potential bias and influence during analysis and selection of data for presentation?* | n | n |
| **IS THERE A CLEAR STATEMENT OF FINDINGS?** | | |
| *Are the findings explicit?* | n | y |
| *Is there adequate discussion of the evidence both for and against the researchers’ arguments?* | n | n |
| *Did the researcher disuccess the credibility ? (e.g. triangulation, respondent validation, more than one analyst.)* | n | n |
| *Are the findings discussed in relation to the original research question?* | n | y |
| **HOW VALUABLE IS THE RESEARCH?** | | |
| *Did the researcher discusses the contribution the study makes to existing knowledge or understanding e.g. do they consider the findings in relation to current practice or policy, or relevant research-based literature?* | n | n |
| *Did they identify new areas where research is necessary?* | y | n |
| *Did the researchers discuss whether or how the findings can be transferred to other populations or considered other ways the research may be used?* | n | y |
| **Questionnaire Quality Assessment** | | |
| **APPROPRIATE SAMPLING?** | | |
| *Was the sampling frame sufficiently large?* | y | y |
| *Was the sampling frame sufficiently representative?* | y | y |
| *Did all participants in the sample understand what was required of them?* | u | u |
| *Did all participants in the sample attribute the same meaning to the terms in the questionnaire?* | u | u |
| **APPROPRIATE INSTRUMENT?** | | |
| *Are there any claims for reliability?* | n | n |
| *Are claims for reliability justified?* | n/a | n/a |
| *Are there any claims for validity?* | n | n |
| *Are claims for validity justified?* | n/a | n/a |
| *Did the questions cover all relevant aspects of the problem?* | u | u |
| *Were questions presented in a non-threatening and non-directive way?* | u | u |
| *Were open-ended (qualitative) used appropriately?* | u | u |
| *Were closed ended (quantitative) questions used appropriately?* | u | u |
| *Was a pilot version administer to participants’ representative of those in the sampling frame?* | y | n |
| *Following piloting, was the instrument modified accordingly if required if required?* | u | n/a |
| **APPROPRIATE RESPONSE?** | | |
| *Was satisfactory response rate achieved?* | y | n |
| *Have non-responders been accounted for?* | n | n |
| **APPROPRIATE CODING AND ANALYSIS?** | | |
| *Was the analysis appropriate (e.g. statistical analysis for quantitative answers, qualitative analysis for open-ended questions)?* | u | u |
| *Were the correct techniques used?* | u | u |
| *Were adequate measures in place to maintain accuracy of data?* | u | u |
| **APPROPRIATE PRESENTATION OF RESULTS?** | | |
| *Have all relevant results (“significant” and “non-significant”) been reported?* | u | u |
| *Was data dredging avoided (i.e. analyses that were not ‘hypothesis driven’)?* | n/a | n/a |
| **RESULTS** | | |
| **MMAT** |  |  |
| Applicable Questions | 5 | 5 |
| Max Score | 10 | 10 |
| Score | 4 | 7 |
| Percentage | 40.00 | 70.00 |
| **CASP** |  |  |
| Applicable Questions | 27 | 28 |
| Max Score | 54 | 56 |
| Score | 8 | 14 |
| Percentage | 14.81 | 25.00 |
| **Questionnaire** |  |  |
| Applicable Questions | 18 | 17 |
| Max Score | 36 | 34 |
| Score | 8 | 4 |
| Percentage | 22.22 | 11.76 |
| **Final Results** |  |  |
| Quality Assessment based on lowest methodological score | 32 (MMAT + CASP) | 22 (MMAT + Q'airre) |
| Max Score | 64 | 44 |
| Score | 12 | 11 |
| Percentage | 18.75 | 25.00 |

*peer-reviewed journal paper, ^conference abstract, y = yes (Score of 2), p = partially (Score of 1) n = no (Score of 0), u = unclear (Score of 0)
