# Supplementary material for: Factors influencing national implementation of innovations within community pharmacy: a systematic review applying the Consolidated Framework for Implementation Research
Source: Implement Sci. 2019 Mar 4;14:21. doi: 10.1186/s13012-019-0867-5 (PMC6398232; doi:10.1186/s13012-019-0867-5)
Supplement: Supplementary file 5 — Full presentation of constructs and subconstructs. (DOCX 536 kb) [file 13012_2019_867_MOESM5_ESM.docx]

Additional file 5: Full presentation of constructs and sub-constructs.

| **CFIR Constructs** | **Sub-constructs** | | | | | |
| --- | --- | --- | --- | --- | --- | --- |
|  | **Barriers** | **N** | **Facilitators** | **N** | **Hypothetical Facilitators** | **N** |
| **Intervention Characteristics** | | | | |  |  |
| A. Intervention Source | Nil | - | Nil | - | Nil | - |
| B. Evidence Strength & Quality | Lack of evidence base (1) | 1 | Nil | - | Nil | -- |
| C. Relative Advantage | Disadvantages of the innovation (2-7) | 6 | Advantages of the innovation (5-16) | 12 | Nil | - |
|  | Lack of observability (17) | 1 |  |  |  |  |
| D. Adaptability | Lack of adaptability (i.e. over restrictive/rigid) (1, 3, 6, 12, 16, 18, 19) | 7 | Adaptability of innovation (19) | 1 | Suggested adaptations to innovation (3, 20) | 2 |
| E. Trialability | Nil | - | Nil | - | Nil | - |
| F. Complexity | Difficulty of innovation (4, 8, 17, 19-21) | 6 | Ease of innovation (7, 21) | 2 | Simplify innovation or make easier (15, 22, 23) | 3 |
|  | Complexity of innovation (15, 17, 19, 20, 22-25) | 8 |  |  |  |  |
|  | Difficulty of implementation innovation (26) | 1 |  |  |  |  |
| G. Design Quality & Packaging | Poor design of innovation (2, 6, 20, 23, 27, 28) | 6 | Convenience of intervention (5, 21) | 2 | Suggested improvements to design/ quality (2, 5, 15, 22-24, 27-31) | 11 |
|  | Poor quality of innovation (6, 7, 10, 19, 28, 29) | 6 |  |  |  |  |
| H. Cost | Increased cost, financial loss or commercial risk relating to innovation (2, 4, 6, 8-10) | 6 | Nil | - | Nil | - |
| **Outer Setting** | | | | |  |  |
| A. Patient Needs & Resources | Patient reluctance or negative views (1, 4, 8, 12, 17, 32) | 6 | Patient acceptance or positive views (9, 11-14, 20, 21, 33) | 8 | Nil | - |
|  | Lack of patient demand (3, 12, 17, 19, 21, 25, 27, 30, 31) | 9 | Patient demand (2, 21) | 2 |  |  |
|  | Lack of patient awareness or knowledge (4, 5, 9, 10, 12, 19, 21, 33) | 8 | Patient awareness (21) | 1 |  |  |
|  | Difficulties recruiting patients or patients non-attendance (6, 10, 12, 19, 20, 34) | 6 | Good relationship between pharmacy and patient (2) | 1 |  |  |
|  | Cost to patients (1, 17, 31) | 3 |  |  |  |  |
|  | Patient’s misuse of service (6) | 1 |  |  |  |  |
|  | Little or no feedback from patients (21) | 1 |  |  |  |  |
|  | Difficulty providing innovation to non-regular patients (27) | 1 |  |  |  |  |
| B. Cosmopolitanism | Negative views of other healthcare professionals (8, 12, 17, 19, 25, 32, 35) | 7 | Referral from other healthcare professionals (3) | 1 | Better engagement or collaboration (5, 25, 29, 32, 36) | 5 |
|  | Lack of healthcare professionals referral or engagement (2, 9, 12, 28) | 4 | Having relationship with other healthcare professionals (9, 12) | 2 |  |  |
|  | Lack of HCP communication/collaboration (6, 12, 16, 17, 19, 24, 37) | 7 | Support from external stakeholders (32) | 1 |  |  |
|  | Other healthcare professionals lack of knowledge (5) | 1 |  |  |  |  |
|  | Poor relationship with other organisations (24) | 1 |  |  |  |  |
| C. Peer Pressure | Nil | - | Nil | - | Seeing colleagues doing it (22) | 1 |
| D. External Policy & Incentives | Lack of, or isufficient funding or remuneration (6, 8, 14, 25, 38) | 5 | Financial incentives (2, 25) | 2 | Financial compensation and incentives (8, 15, 22-24, 27, 30, 32, 38) | 9 |
|  | Innovation not being policy (27) | 1 |  |  | Making the innovation compulsory (15, 22-24, 29) | 5 |
|  |  |  |  |  | Increasing scope of the innovation (25, 27, 32) | 3 |
|  |  |  |  |  | Apply penalties to non-compliant physicians | 1 |
| **Inner Setting** |  |  |  |  |  |  |
| A. Structural Characteristics | Nil | - | Small pharmacy with few staff (3) | 1 | Nil | - |
|  |  |  | Rurality of pharmacy meaning have previous acquaintances with patients (21) | 1 |  |  |
|  |  |  | Well organised workflow systems (19) | 1 |  |  |
| B. Networks & Communication | Lack of communication within pharmacy (3, 21) | 2 | Teamwork and communication within pharmacy (21) | 1 | Nil | - |
|  |  |  | Alignment of innovation with the values of pharmacy team (39) | 1 |  |  |
| C. Culture | Nil | - | Nil | - | Nil | - |
| D. Implementation Climate | | | | | | |
| *i. Tension for change* | Organisational culture accepting change (19) | 1 | Nil | - |  | - |
| *ii. Compatibility* | Fear of, or increased, legal liability (8, 15, 22-24) | 5 | Compatibility of innovation with roles or values (10, 15-17, 19, 20, 22-24, 36) | 10 | Suggested workflow changes (27) | 1 |
|  | Incompatibility of innovation with pharmacy setting or processes (21, 27) | 2 | Compatibility of innovation with working systems (19, 21, 37) | 3 |  |  |
|  | Incompatibility of innovation with wider healthcare service (16, 19) | 2 |  |  |  |  |
|  | Innovation outwith pharmacy remit (21) | 1 |  |  |  |  |
| *iii. Relative Priority* | Competing priorities (18, 21, 24) | 3 | Nil | - | Nil | - |
| *iv. Organisation Incentives & Rewards* | Target setting relating to innovation perceived as income focused and not based on patient needs (16) | 1 | Improved professional recognition, influence, or extended professional role (6, 8, 10, 12, 17, 20, 30, 31) | 8 |  |  |
|  |  |  | Commercial benefits or increased footfall in pharmacy (5, 6, 10, 14, 17, 21, 32) | 7 |  |  |
|  |  |  | Professional satisfaction (19, 20, 32, 33, 38) | 5 |  |  |
| *v. Goals and Feedback* | Lack of feedback in pharmacy (37) | 1 | Recieving feedback (6, 39) | 2 | Receiving feedback (15, 18, 22-24, 29) | 6 |
|  | Lack of feedback from external organisations (29) | 1 |  |  |  |  |
| *vi. Learning Climate* | Nil | - | Nil | - | Nil | - |
| E. Readiness for Implementation | | | | | | |
| *i. Leadership engagement* | Lack of leadership engagement (24, 37) | 2 | Leadership engagement (21, 39) | 2 | Nil | - |
|  | Lack of leadership skills (37) | 1 | Pharmacists leadership (21) | 1 |  |  |
|  | Reliance on pharmacist leadership (21) | 1 |  |  |  |  |
| *ii. Available Resources* | Time constraints or increased workload (6-11, 14-16, 18, 21-27, 29, 30, 32, 34-38) | 25 | Valued resources (3, 5, 10, 19, 21, 37) | 6 | Suggested resources (2, 6, 7, 16, 22, 25-27, 30) | 9 |
|  | Lack of resources (6, 15, 18, 22-24, 27, 29) | 8 | Suitable spaces to counsel patients (35, 37) | 2 | Better support (26, 33) | 2 |
|  | Lack of access to clinical information about patients (1, 6, 17, 28, 30) | 5 | Having two pharmacists on duty (19) | 1 |  |  |
|  | Staffing issues (10, 17, 30, 35, 38) | 5 | Support from professional body (17) | 1 |  |  |
|  | Lack of space or suitable area (17, 21, 30, 38) | 4 |  |  |  |  |
|  | Lack of support (9) | 1 |  |  |  |  |
| *Iii. Access to Knowledge and Information* | Lack of appropriate training about innovation (2, 16, 21, 31, 37) | 5 | Access to information or being well informed about innovation (3, 17, 21, 37) | 4 | Better training or access to information about innovation (2, 3, 8, 12, 13, 15, 19, 21-24, 26, 27, 29, 36-38) | 17 |
|  | Lack of information about innovation (6, 19, 21) | 3 | Good training (10, 21) | 2 |  |  |
| **Characteristics of Individuals** | |  |  |  |  |  |
| A. Knowledge and Beliefs about the intervention | Negative pharmacy staff views about the innovation (1-3, 13, 15-17, 19, 21-24, 29-32, 35, 39) | 18 | Positive pharmacy staff views about the innovation (2, 3, 8, 10, 11, 15, 17, 20-26, 28-32, 35, 36) | 21 | Nil | - |
| - | Lack of pharmacy staff awareness or knowledge (3, 15, 21-24, 26, 29, 36, 38) | 10 | Pharmacy staff awareness or knowledge about the innovation (3, 15, 32, 36) | 4 |  |  |
|  | Lack of pharmacy staff’s clinical knowledge (15, 18, 22, 23, 29) | 5 |  |  |  |  |
| B. Self-Efficacy | Lack of pharmacy staff’s confidence (16, 18, 33) | 3 | Confidence of pharmacy staff (13, 17, 26, 31, 33) | 5 | Nil | - |
|  | Belief that skills cannot be developed (39) | 1 | Belief that success of programme influenced by own approach (39) | 1 |  |  |
| C. Individual Stage of Change | Reluctance or lack of motivation regarding innovation (3, 8, 15, 22, 23, 27) | 6 | Willingness or enthusiasm regarding innovation (3, 9, 13, 14, 21, 32, 33, 37, 39) | 9 | Nil | - |
| D. Individual Identification with Organisation | Nil | - | Nil | - | Nil | - |
| E. Other Personal Attributes | Innovation not aligning with personal gains (15, 22, 23) | 3 | Pharmacy staff having self-resilience when experience negative feedback (21) | 1 | Nil | - |
|  | Pharmacy staff having a dispensing-focused role (21) | 1 | Good communication skills of pharmacy staff (21) | 1 |  |  |
|  |  |  | Pharmacy staff working >21 hours a week (30) | 1 |  |  |
|  |  |  | The pharmacist being store based and not a locum pharmacist (30) | 1 |  |  |
|  |  |  | Having higher socio-economic clientele in pharmacy (3) | 1 |  |  |
|  |  |  | Pharmacy staff having altruistic personality (32) | 1 |  |  |
|  |  |  | Being a younger pharmacist (6) | 1 |  |  |
|  |  |  | Pharmacy staff being based at front counter (21) | 1 |  |  |
|  |  |  | Pharmacy staff having previous experience (21) | 1 |  |  |
| **Process** |  |  |  |  |  |  |
| A. Planning | No piloting and evaluation of innovation before national implementation (19) | 1 | Nil | - | Better piloting and planning of innovation (19) | 1 |
|  |  |  |  |  | More methodical implementation plan for innovation (24) | 1 |
|  |  |  |  |  | Development of clear aims and objectives (21) | 1 |
| B. Engaging Stakeholders | Lack of undergraduate exposure to innovation (23) | 1 | Nil | - | Better promotions or engagement with pharmacies (2, 9, 15, 19, 22-24, 36, 37) | 9 |
|  |  |  |  |  | Informing/engaging other HCPs (5, 8, 12, 19, 36) | 5 |
| C. Engaging Intervention Participants | Lack of advertising or promotion of innovation(6, 19, 23) | 3 | Customer awareness of innovation though use of banners and displays (14) | 1 | Better informing or educating the public about innovation (5, 6, 8-10, 13, 15, 21, 25, 27, 32, 36) | 12 |
| D. Executing | Nil | - | Nil | - | Nil | - |
| E. Reflecting and Evaluating | Nil | - | Nil | - | Ongoing review by commissioners once innovation implemented (19) | 1 |

**References**

1. Hansford D, Cunningham S, John D, McCaig D, Stewart D. Community pharmacists’ views, attitudes and early experiences of over-the-counter simvastatin. Pharm World Sci. 2007;29(4):380-5.

2. Chaar BB, Wang H, Day CA, Hanrahan JR, Winstock AR, Fois R. Factors influencing pharmacy services in opioid substitution treatment. Drug Alcohol Rev. 2013;32(4):426-34.

3. Gauld N, Kelly F, Shaw J. Is non-prescription oseltamivir availability under strict criteria workable? A qualitative study in New Zealand. J Antimicrob Chemother. 2011;66(1):201-4.

4. Gröber-Grätz D, Gulich M. Impact of drug discount contracts on pharmacies and on patients’ drug supply. J Public Health. 2010;18(6):583-9.

5. Hammar T, Nystrom S, Petersson G, Rydberg T, Astrand B. Swedish pharmacists value eprescribing: A survey of a nationwide implementation. J Pharm Health Serv Res. 2010;1(1):23-32.

6. Paudyal V, Hansford D, Scott Cunningham IT, Stewart D. Cross-sectional survey of community pharmacists' views of the electronic Minor Ailment Service in Scotland. Int J Pharm Pract. 2010;18(4):194-201.

7. Rahimi B, Timpka T. Pharmacists' views on integrated electronic prescribing systems: associations between usefulness, pharmacological safety, and barriers to technology use. Eur J Clin Pharmacol. 2011;67(2):179-84.

8. Allenet B, Barry H. Opinion and behaviour of pharmacists towards the substitution of branded drugs by generic drugs: survey of 1,000 French community pharmacists. Pharm World Sci. 2003;25(5):197-202.

9. Brooks D, Hopp A, White S. Perspectives of community pharmacy staff on Healthy Living Pharmacies: A qualitative study. Int J Clin Pharm. 2013;21(Suppl. 2):16.

10. Firth H, Todd A, Bambra C. Benefits and barriers to the public health pharmacy: a qualitative exploration of providers’ and commissioners’ perceptions of the Healthy Living Pharmacy Framework. Perspect Public Health. 2015;135(5):251-6.

11. Lonergan C, O'Grady M, Byrne S. An exploratory study of codeine sales restrictions within Irish pharmacies, a qualitative study. Int J Pharm Pract. 2012;20(Suppl. 1):43.

12. Lucas B, Blenkinsopp A. Community pharmacists' experience and perceptions of the New Medicines Service (NMS). Int J Pharm Pract. 2015;23(6):399-406.

13. Ping CC, March G, Clark A, Gilbert A, Hassali MA, Bahari MB. A Web-Based Survey on Australian Community Pharmacists' Perceptions and Practices of Generic Substitution. J Generic Med. 2010;7(4):342-53.

14. Shevket O, White S. A qualitative follow-up study of the perspectives of community pharmacy staff on Healthy Living Pharmacies. Int J Clin Pharm. 2015;23(Suppl. 2):69-70.

15. Van Grootheest AC, den Berg LTWdJ-v, Mes K. Attitudes of community pharmacists in the Netherlands towards adverse drug reaction reporting. Int J Pharm Pract. 2002;10(4):267-72.

16. Wilcock M, Harding G. What do pharmacists think of MURs and do they change prescribed medication? Pharm J. 2007;281:163.

17. Paudyal V, Hansford D, Cunningham S, Stewart D. Pharmacists' perceived integration into practice of over-the-counter simvastatin five years post reclassification. Int J Clin Pharm. 2012;34(5):733-8.

18. Kaae S, Sondergaard B, Stig L, Traulsen JM. Sustaining delivery of the first publicly reimbursed cognitive service in Denmark: A cross-case analysis. Int J Pharm Pract. 2010;18(1):21-7.

19. Latif A, Waring J, Watmough D, Barber N, Chuter A, Davies J, et al. Examination of England's New Medicine Service (NMS) of complex health care interventions in community pharmacy. Res Social Adm Pharm. 2016;12(6):966-89.

20. Corlett S, Dodds L. The New Medicines Service; Initial views and early experiences of Community Pharmacists in Kent. Int J Clin Pharm. 2013;21(Suppl. 2):96-7.

21. Donovan GR, Paudyal V. England's Healthy Living Pharmacy (HLP) initiative: Facilitating the engagement of pharmacy support staff in public health. Res Social Adm Pharm. 2016;12(2):281-92.

22. Bawazir S. ATTITUDE OF COMMUNITY PHARMACISTS IN SAUDI ARABIA TOWARDS ADVERSE DRUG REACTION REPORTING. Saudi Pharm J. 2006;14(1):75-83.

23. Elkalmi RM, Hassali MA, Ibrahim MI, Jamshed SQ, Al-Lela OQ. Community pharmacists' attitudes, perceptions, and barriers toward adverse drug reaction reporting in Malaysia: a quantitative insight. J Patient Saf. 2014;10(2):81-7.

24. Elkalmi RM, Hassali MA, Ibrahim MIM, Liau SY, Awaisu A. A qualitative study exploring barriers and facilitators for reporting of adverse drug reactions (ADRs) among community pharmacists in Malaysia. J Pharm Health Serv Res. 2011;2(2):71-8.

25. Lee E, Braund R, Tordoff J. Examining the first year of Medicines Use Review services provided by pharmacists in New Zealand: 2008. N Z Med J. 2009;122(1293):3566.

26. Thomas T, Hathiari A, Benson C, Oladosu B. Controlled drugs: are they being controlled? An evaluation of the views, understanding and implementation of the amended Controlled Drug Regulations (2006) by community pharmacists. Int J Pharm Pract. 2009;17(Suppl. 2):75.

27. Hamrosi KK, Raynor DK, Aslani P. Enhancing provision of written medicine information in Australia: pharmacist, general practitioner and consumer perceptions of the barriers and facilitators. BMC Health Serv Res. 2014;14(1):183.

28. Hodson K, James D, Smith M, Hughes L, Blenkinsopp A, Cohen D, et al. Evaluation of the Discharge Medicines Review Service in Wales: community and hospital pharmacists’ views. Int J Clin Pharm. 2014;22(Suppl. 2):6.

29. Duarte M, Ferreira P, Soares M, Cavaco A, Martins AP. Community pharmacists’ attitudes towards adverse drug reaction reporting and their knowledge of the new pharmacovigilance legislation in the southern region of Portugal: a mixed methods study. Drugs Ther Perspect. 2015;31(9):316-22.

30. Latif A, Boardman H. Community pharmacists' attitudes towards medicines use reviews and factors affecting the numbers performed. Pharm World Sci. 2008;30(5):536-43.

31. Weidmann AE, Cunningham S, Gray G, Hansford D, McLay J, Broom J, et al. Over-the-counter orlistat: early experiences, views and attitudes of community pharmacists in Great Britain. Int J Clin Pharm. 2011;33(4):627-33.

32. Bell CA, Eang MT, Dareth M, Rothmony E, Duncan GJ, Saini B. Provider perceptions of pharmacy-initiated tuberculosis referral services in Cambodia, 2005-2010. Int J Tuberc Lung Dis. 2012;16(8):1086-91.

33. Rutter P, Vryaparj G. Qualitative exploration of the views of healthy living champions from pharmacies in England. Int J Clin Pharm. 2015;37(1):27-30.

34. Blenkinsopp A, Celino G, Bond C, Inch J, Gray N. Community pharmacists’ experience of providing medicines use reviews: findings from the national evaluation of the community pharmacy contractual framework. Int J Clin Pharm. 2007;15(Suppl. 2):845-6.

35. Latif A, Mahmood K, Boardman H. Medicines Use Reviews – how have pharmacists’ views changed? Int J Clin Pharm. 2010;18(Suppl. 2):69-70.

36. Irujo M, Beitia G, Bes-Rastrollo M, Figueiras A, Hernandez-Diaz S, Lasheras B. Factors that influence under-reporting of suspected adverse drug reactions among community pharmacists in a Spanish region. Drug Saf. 2007;30(11):1073-82.

37. Kansanaho H, Puumalainen I, Varunki M, Ahonen R, Airaksinen M. Implementation of a professional program in Finnish community pharmacies in 2000-2002. Patient Educ Couns. 2005;57(3):272-9.

38. Loo RL, Diaper C, Salami OT, Kundu M, Lalkia M, Airhiavbere E, et al. The NHS Health Check: The views of community pharmacists. Int J Pharm Pract. 2011;19:13.

39. Kaae S, Søndergaard B, Haugbølle LS, Traulsen JM. The relationship between leadership style and provision of the first Danish publicly reimbursed cognitive pharmaceutical service—A qualitative multicase study. Res Social Adm Pharm. 2011;7(2):113-21.
