# Supplementary material for: Factors influencing national implementation of innovations within community pharmacy: a systematic review applying the Consolidated Framework for Implementation Research
Source: Implement Sci. 2019 Mar 4;14:21. doi: 10.1186/s13012-019-0867-5 (PMC6398232; doi:10.1186/s13012-019-0867-5)
Supplement: Supplementary file 6 — CFIR constructs represented within the different reviews. (DOCX 33 kb) [file 13012_2019_867_MOESM6_ESM.docx]

Additional file 6: CFIR constructs represented within the different reviews.

| **CFIR Constructs** | **Current review** | **Shoemaker et al’s review [1]** | | | **Robert et al’s review^ [2]** |
| --- | --- | --- | --- | --- | --- |
|  | National innovations | MTM | Immuniz-ations | HIV testing | CPS |
| **Intervention Characteristics** |  |  |  |  |  |
| Intervention Source | - | - | - | - | - |
| Evidence Strength & Quality | ✔ | ✔ | - | - | - |
| Relative Advantage | ✔ | ✔ | ✔ | ✔ | ✔ |
| Adaptability | ✔ | ✔ | ✔ | ✔ | - |
| Trialability | - | - | ✔ | - | - |
| Complexity | ✔ | ✔ | ✔ | - | - |
| Design Quality & Packaging | ✔ | - | - | ✔ | - |
| Cost | ✔ | ✔ | ✔ | ✔ | - |
| **Outer Setting** |  |  |  |  |  |
| Patient Needs & Resources | ✔ | ✔ | ✔ | ✔ | ✔ |
| Cosmopolitanism | ✔ | - | ✔ | ✔ | ✔ |
| Peer Pressure | ✔ | ✔ | - | ✔ | - |
| External Policy & Incentives | ✔ | ✔ | ✔ | ✔ | ✔ |
| **Inner Setting** |  |  |  |  |  |
| Structural Characteristics | ✔ | ✔ | ✔ | ✔ | ✔ |
| Networks & Communications | ✔ | - | - | - | ✔ |
| Culture | - | ✔ | - | ✔ | ✔ |
| Implementation Climate | ✔ | ✔ | ✔ | ✔ | ✔ |
| *Tension for Change* | *✔* | *** | *** | *** | *-* |
| *Compatibility* | *✔* | *** | *** | *** | *-* |
| *Relative Priority* | *✔* | *** | *** | *** | *-* |
| *Organizational Incentives & Rewards* | *✔* | *** | *** | *** | *✔* |
| *Goals and Feedback* | *✔* | *** | *** | *** | *-* |
| *Learning Climate* | *-* | *** | *** | *** | *-* |
| Readiness for Implementation | ✔ | ✔ | ✔ | ✔ | ✔ |
| *Leadership Engagement* | *✔* | *** | *** | *** | *✔* |
| *Available Resources* | *✔* | *** | *** | *** | *✔* |
| *Access to Knowledge & Information* | *✔* | *** | *** | *** | *✔* |
| **Characteristics of Individuals** |  |  |  |  |  |
| Knowledge & Beliefs about the Intervention | ✔ | ✔ | ✔ | ✔ | ✔ |
| Self-efficacy | ✔ | ✔ | ✔ | - | ✔ |
| Individual Stage of Change | ✔ | - | - | - | ✔ |
| Individual Identification with Organization | - | - | - | - | - |
| Other Personal Attributes | ✔ | ✔ | - | - | ✔ |
| **Process** |  |  |  |  |  |
| Planning | ✔ | ✔ | ✔ | - | - |
| Engaging | ✔ | ✔ | ✔ | ✔ | ✔ |
| *Engaging (Stakeholders)* | ✔ | * | * | * | - |
| *i. Opinion Leaders* | - | * | * | * | - |
| *ii. Formally Appointed Internal Opinion Leaders* | - | * | * | * | - |
| *iii. Champions* | - | * | * | * | ✔ |
| *iv. External Change Agents* | - | * | * | * | ✔ |
| *Engaging (Innovation Participants)* | ✔ | * | * | * | ✔ |
| Executing | ✔ | ✔ | ✔ | - | - |
| Reflecting & Evaluating | ✔ | - | ✔ | - | - |

MTM – medication therapy management, CPS - cognitive pharmacy services,✔Represents that the CFIR construct was observed within the literature, - Represents that a CFIR construct was not observed within the literature, ^Mapping of the facilitators identified by Roberts et al review is available upon request, *As the tabulation of results by Shoemaker et als study was based on the overarching construct and not the sub-construct as with the current review (e.g. “Implementation Climate” and not “Tension for Change”, “Compatibility” etc.) cross comparison not possible

Individual facilitators Organisational facilitators

• Pharmacist competence

28,34

• Physical environment e.g. adequate space/privacy and workflow

16,22,28,30,34,35,37,58–60

• Education and training for pharmacy assistants

40,56,57

• Culture of the pharmacy

35,61

• Education and training for pharmacists

16,19,22,23,30,37,40,57–59

• Remuneration/incentives

14,16,19,34,35,57–59

• Communication skills

22,32

• Sufficient and qualified staff/manpower

28,30

• Motivation

37,57

• Use of pharmacy technicians

2,15,36,40,58

• Leadership skills

30

• Delegation of tasks

37,58

• Professional satisfaction

24,39

• Innovative practice orientation

26,28

• Pharmacists’ knowledge of CPS

19

• Patient demand/expectations

15,28,59

• Pharmacists’ attitudes towards CPS

22,33,34,59

• Relationship with doctors

22,28,30,34,35,58

• Pharmacists’ confidence in ability to provide CPS

33

• Equipment and technology, e.g. computers

2,15,22,28,30

• Autonomy

23

• Access to patient information/records

22,28,34

• Attitude of pharmacy staff

16

• Documentation system

32,35,58

• Profile within the local community

40

• Attention for special patient groups

37

• Use of protocols

37,59

• Interaction with other pharmacists

16,23,34,56

• Support of management

40,61

• Access to reference literature

35

• Pharmacist–patient relationship

56,58

• Marketing

18,37,59

• Support from professional organisations and/or government

16,30,34,58

• Low script volume

32

• Rural location

32

• Legislation requiring or supporting provision of services

16,19,22,35,42

• Attitude/perception of doctors

22

• Attitude perception of patients

34,57

• Examples from leading practitioners

16,59

• External advisors or mentors

23,59

• Evidence of benefits of services

22,35

**References**

1. Shoemaker SJ, Curran GM, Swan H, Teeter BS, Thomas J. Application of the Consolidated Framework for Implementation Research to community pharmacy: A framework for implementation research on pharmacy services. Res Social Adm Pharm. 2017;13:905-13. doi: 10.1016/j.sapharm.2017.06.001.

2. Roberts AS, Benrimoj SI, Chen TF, Williams KA, Aslani P. Implementing cognitive services in community pharmacy: a review of facilitators used in practice change. Int J Pharm Pract. 2006;14:163-70. doi: 10.1211/ijpp.14.3.0002.
